# Occurrence and Detection Profiles of Technology-Critical Elements (Ga, Ge, Nb, Ta and W) in Wild Mushrooms from Leicester and Leicestershire, UK

**DOI:** 10.3390/toxics14080723

**Published:** 2026-08-14

**Authors:** Antonio Peña-Fernández, Tomás Cámara-Pastor, Guillermo Torrado Durán, M. Carmen Lobo-Bedmar

**Affiliations:** 1 Area of Legal and Forensic Medicine, Department of Surgery, Medical and Social Sciences, Faculty of Medicine and Health Sciences, University of Alcalá, Ctra. Madrid-Barcelona Km, 33.6, 28871 Alcalá de Henares, Spain; 2 Leicester School of Allied Health Sciences, De Montfort University, Leicester LE1 9BH, UK; 3 Scientific Computation Research Institute (SCRIUR), University of La Rioja, 26006 Logroño, Spain; 4 Department of Biomedical Sciences, Faculty of Pharmacy, University of Alcalá, Crta. Madrid-Barcelona Km, 33.6, 28871 Alcalá de Henares, Spain; 5 Departamento de Investigación Agroambiental, Madrid Institute for Rural, Agricultural and Food Research and Development (IMIDRA), Finca el Encín, Crta. Madrid-Barcelona Km, 38.2, 28800 Alcalá de Henares, Spain

**Keywords:** wild mushrooms, technology-critical elements, emerging environmental contaminants, gallium, germanium, niobium, tantalum, tungsten, fungal bioindication, environmental monitoring, ICP-MS, censored data

## Abstract

Technology-critical elements are increasingly used in electronics, renewable-energy systems, high-performance alloys and other strategic technologies, yet their occurrence in wild fungi remains poorly characterised. This study evaluated gallium (Ga), germanium (Ge), niobium (Nb), tantalum (Ta) and tungsten (W) in wild mushrooms collected from Leicester and Leicestershire, UK. The dataset was reconstructed at fruiting-body level to avoid pseudo-replication from paired cap and stem records, yielding 121 analytical units. Results were corrected using digestion-date acid blanks, interpreted against analytical-portion-specific working limits of detection and classified as fully quantified (FQ), partially censored (PC) or fully censored (FC). Ga was FQ in 106/121 units and was the only element with sufficient quantitative coverage for continuous distributional estimation; its regression-on-order-statistics median was 475.48 ng g^−1^ dw (IQR: 278.70–941.63). The corresponding FQ counts were 16 for Ge, 33 for Nb, 10 for Ta and 24 for W, with 7, 4 and 4 additional PC observations for Nb, Ta and W, respectively. In 22 paired *Agaricus bitorquis* fruiting bodies, Ga concentrations were higher in stems than caps (Wilcoxon *p* = 5.25 × 10^−5^; BH-FDR = 1.05 × 10^−4^). Composite topsoils from 26 monitored sites provided park-scale geochemical context only and were not used to infer bioaccumulation or direct soil-to-fungus transfer. These findings establish a blank-corrected and censoring-aware occurrence baseline and suggest exploratory, site-specific potential for wild mushrooms as complementary biomonitoring matrices for selected technology-critical elements, while demonstrating that their usefulness is element-, taxon- and design-dependent.

## 1. Introduction

Technology-critical elements (TCEs) are increasingly used in semiconductors, optoelectronics, photovoltaic technologies, fibre optics, capacitors, hard metals, superalloys and other components supporting the green and digital transitions [1,2,3]. Their strategic importance is reflected in the European Union Critical Raw Materials framework and Regulation (EU) 2024/1252, which established measures intended to secure a sustainable supply of critical and strategic raw materials [2,4]. Gallium (Ga), germanium (Ge) and tungsten (W) are included among the strategic raw materials, while niobium (Nb) and tantalum (Ta) form part of the broader critical-raw-materials framework. This policy classification is not a toxicological ranking. Nevertheless, expanding production, use, recycling and waste flows may increase opportunities for environmental release. Their growing technological use, limited routine environmental surveillance and incompletely characterised occurrence and behaviour support their consideration within the broader field of emerging environmental contaminants.

Ga and Ge are widely associated with semiconductor, optoelectronic, photovoltaic, fibre-optic and infrared applications [1,3]. Their environmental behaviour is strongly dependent on matrix, speciation, geochemical conditions and biological availability [3,5,6,7,8]. At continental scale, Ga distribution in European agricultural soils has been associated with parent material, weathering and provenance, illustrating the importance of the local geochemical setting [8]. Nb and Ta are high-field-strength elements commonly associated with resistant mineral phases, low mobility and strong lithogenic control, although biological uptake has been reported under particular environmental conditions [9,10,11,12]. W has historically been used in hard metals, alloys, military materials and industrial applications, and its environmental mobility can be affected by tungstate speciation, pH, soil composition, microbial activity and other local conditions [13,14,15,16,17,18,19]. Despite their increasing use, these elements remain much less routinely monitored than established contaminants such as Cd, Pb, Hg or As.

Wild mushrooms are valuable biological matrices for environmental monitoring because their fruiting bodies integrate influences from the substrate, mycelial activity, fungal taxonomy, trophic strategy and local microhabitat [20,21,22,23,24]. They should not, however, be regarded as passive reflections of bulk soil composition or as universally efficient accumulators of every element. Mushroom analysis can document environmental occurrence and relative fruiting-body patterns, but it cannot by itself demonstrate contaminant sources, biological effects on fungi or toxicological risk. Although wild mushrooms have been widely studied for classical metal(loid)s and several less routinely monitored elements, field data specifically addressing Ga, Ge, Nb, Ta and W remain scarce [20,21,22,23,24,25,26,27,28,29,30,31]. Existing multielement studies have occasionally reported one or more of these elements in road-influenced wild mushrooms and in taxa including *Macrolepiota procera*, *Amanita muscaria* and *Leccinum scabrum* [27,29,30,31]. Experimental studies have also shown that cultivated mushrooms can accumulate Ge following substrate supplementation, but such controlled observations cannot be directly extrapolated to naturally occurring wild fungi [32].

From a toxicological perspective, the occurrence of technology-critical elements in wild mushrooms is relevant because selected fungal taxa may be collected for human consumption, thereby providing a potential dietary exposure pathway. However, characterising the toxicological risk associated with Ga, Ge, Nb, Ta and W in wild edible mushrooms is particularly difficult. Total dry-weight concentrations alone do not define the ingested or systemically available dose. A defensible assessment would additionally require taxon-specific consumption frequencies and portion sizes, fresh-to-dry-weight conversion, the effects of washing and culinary processing, gastrointestinal bioaccessibility, chemical speciation and information on absorption, distribution, metabolism and elimination. Food-specific health-based guidance values, chronic oral dose–response relationships and toxicokinetic information are comparatively limited for Ga, Ge, Nb, Ta and W relative to more extensively studied foodborne contaminants [1,3,13,20,23]. Potential combined exposure to several technology-critical elements is even less characterised. Baseline occurrence data are therefore necessary for hazard identification and exposure prioritisation, but cannot independently establish either toxicological safety or an unacceptable health risk.

The available fungal literature is geographically and taxonomically uneven, and systematic occurrence data for these less routinely monitored technology-critical elements in terrestrial biota remain limited across much of Europe, including the UK [20,21,22,23,24,25,26,27,28,29,30,31,32]. Leicester and Leicestershire provide a mixed urban–rural framework comprising public parks, redeveloped urban land, transport corridors, waterways, rural green spaces and areas influenced by varied historical land uses [33,34,35,36,37,38]. These locations were not selected because they were known to contain unusually high concentrations of the target elements. Instead, they provided a heterogeneous setting in which to establish baseline occurrence and detection profiles across urban, peri-urban and rural green spaces without presupposing contamination or a particular emission source.

Interpretation of mushroom elemental data requires careful definition of the analytical unit and appropriate treatment of observations below the analytical limit of detection (LoD). Caps and stems are sometimes analysed separately because anatomical partitioning can differ between tissues and elements [22,24,39]. However, treating matched cap and stem measurements from the same fruiting body as independent samples can inflate the apparent sample size and disproportionately weight taxa for which tissue-separated material is available. Reconstruction at fruiting-body level therefore provides a more appropriate unit for overall, taxonomic and spatial summaries. Low-level measurements additionally require explicit consideration of acid-digestion blanks, sample mass, sample-specific LoDs and the proportion of censored observations. Substitution-based summaries can substantially distort distributions when censoring is high; consequently, quantitative descriptors and multivariable analyses should be restricted to elements and subsets with sufficient detection frequencies [40,41].

Composite topsoil data generated within the same field-sampling programme provide additional information on the park-scale geochemical setting [33]. However, the topsoil composites were not collected directly beneath individual fruiting bodies and do not represent mushroom-specific substrates or mycelial microhabitats. They are therefore used only as contextual environmental descriptors. Direct soil-to-fungus transfer, bioaccumulation factors and causal relationships cannot be estimated from these datasets because the soil and mushroom samples are not spatially paired at the necessary biological scale.

Accordingly, the aims of this study were to: (i) determine blank-corrected fruiting-body-level occurrence and detection profiles for Ga, Ge, Nb, Ta and W in wild mushrooms collected from Leicester and Leicestershire; (ii) quantify the extent of censoring and apply reporting approaches appropriate to the detection frequency of each element; (iii) examine taxon-level and cap–stem patterns where analytical detection supported such interpretation; (iv) describe urban–rural and within-urban patterns cautiously, recognising the opportunistic sampling design and differences in taxonomic composition; and (v) contextualise the monitored green spaces using site-level composite topsoil concentrations, comparative geochemical screening ratios based on the pollution index (PI) and Fe-normalised enrichment factor (EF), and exploratory topsoil inter-element relationships. The study was designed as a baseline environmental-occurrence assessment and not as an evaluation of effects on fungi, dietary exposure, toxicological risk or direct soil-to-fruiting-body transfer.

## 2. Materials and Methods

### 2.1. Study Area and Field Sampling Framework

Wild mushroom fruiting bodies were collected from urban green spaces in Leicester and selected rural and semi-rural sites in Leicestershire, UK, as part of a wider field-sampling programme described previously in a doctoral thesis and a peer-reviewed study [33,42]. The published study examined Cd, Cu and Zn, whereas the present investigation evaluates Ga, Ge, Nb, Ta and W measured in the same archived mushroom and composite topsoil sample sets. Mushrooms were the primary biological matrix in this study; composite topsoils were included only to contextualise the monitored green-space geochemical setting. Leicester was treated as the principal urban study area, whereas selected Leicestershire locations represented rural or semi-rural green-space environments outside the main urban boundary.

Leicester provides a relevant urban green-space setting because it is a long-established East Midlands city with a history of industrial development, transport corridors, redeveloped land and public open spaces embedded within the urban fabric. Historical industrial activity in Leicester and the wider county has included hosiery, knitwear, boot and shoe manufacture, engineering, metal working, food processing, electronics, printing and plastics, alongside rural and peri-urban influences such as agriculture, quarrying, brick-making, coalfield activity and rural-industrial legacies [33,34,35,36,37]. These features are relevant as broad contextual factors because legacy manufacturing, road traffic, canal and river corridors, made ground, demolition residues, road-deposited dust and reworked urban fill may contribute to spatially heterogeneous geochemical signatures in urban green-space environments. They were not used to assign element sources to individual mushroom samples.

The wider environmental framework included urban parks, open spaces and rural green areas. These sites provide a mixed public-access landscape influenced by urban development, traffic, land management, local geology, superficial deposits and historical land-use legacies [33,38]. Mushroom collection was conducted across multiple field campaigns and was contingent on the natural occurrence of eligible fruiting bodies at the visited locations, as detailed in Section 2.2.

The associated topsoil programme characterised whole parks and green-space areas using homogenised composite samples. This design provides park-scale geochemical information for the monitored areas but does not represent substrate sampled immediately beneath individual fruiting bodies. Briefly, the wider topsoil framework comprised 26 homogenised site-level composite topsoil samples generated from 850 field increments collected across 18 urban parks/open spaces and eight rural Leicestershire sites [42]. This distinction defines the environmental scale of the study: mushrooms were analysed at fruiting-body level, whereas topsoil data describe the monitored area as a composite geochemical unit.

**Figure 1:**
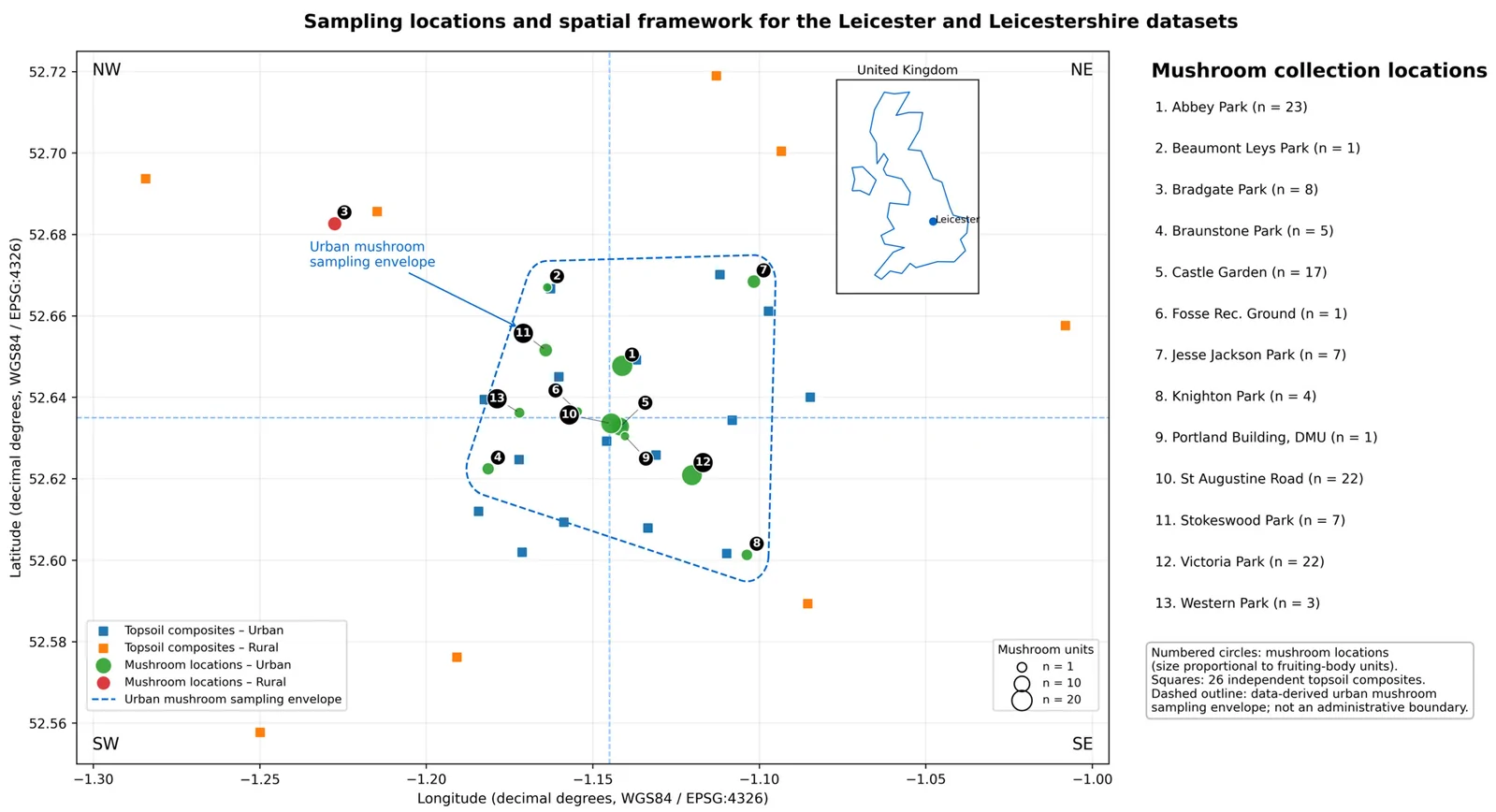
Sampling locations and spatial framework for the Leicester and Leicestershire mushroom and composite topsoil datasets. Numbered circles identify the 13 mushroom collection locations, with symbol size proportional to the number of reconstructed fruiting-body units collected at each location (total *N* = 121). Squares identify the locations represented by the 26 site-level composite topsoil values, and colours distinguish urban and rural locations. The inset shows the location of Leicester within the United Kingdom, while the dashed lines indicate the quadrant framework used for descriptive spatial grouping. The mushroom and topsoil datasets represent different spatial scales and were not paired at individual fruiting-body level. The dashed polygon represents a data-derived envelope around the urban mushroom sampling locations and should not be interpreted as an administrative boundary.

The spatial framework and distribution of the mushroom collection locations and composite topsoil sites are shown in Figure 1.

### 2.2. Wild Mushroom Collection, Labelling and Taxonomic Identification

Wild mushroom field campaigns were conducted on 12 dates between 12 September and 18 November 2019. Sampling was scheduled only after a minimum of 24 h without rainfall, and the recorded ambient temperatures ranged from 8 to 17 °C. Accessible areas of the selected parks and green spaces were searched visually, and the location of each collection point was recorded using a handheld Global Positioning System (GPS) device.

Only fruiting bodies with a fresh, young and intact appearance were eligible for collection. Specimens showing visible decay, insect damage, signs of parasitism, trampling or other physical alteration were excluded. Fruiting bodies growing within or immediately adjacent to visible litter or other anthropogenic waste, or within visibly disturbed microsites, were also not collected.

Sampling was based on the natural availability of fruiting bodies meeting the predefined collection criteria at the locations visited during the field campaigns. The design was intended to characterise elemental occurrence in collected fruiting bodies rather than fungal diversity or prevalence and therefore did not involve systematic coverage of all parks, standardised transects or equal sampling effort across locations. Although sampling across multiple campaign dates extended the temporal coverage, differences in fruiting-body availability and detectability—including the preferential detection of larger or more conspicuous specimens—could not be excluded. Accordingly, the dataset characterises elemental occurrence among specimens meeting the collection criteria and should not be interpreted as a representative survey of fungal community composition or prevalence. No minimum separation between collected fruiting bodies was predefined, and the spatial extent and genotypic identity of the underlying mycelia were not characterised. Fruiting bodies collected within the same location may therefore represent spatially clustered observations and may not correspond to independent fungal genets. The same fungal taxa were not present or collected at all locations; consequently, taxonomic composition and sample size differed among sites and campaign dates, and the dataset was not balanced for direct species-by-location comparisons.

Specimens were handled with nitrile gloves to minimise external contamination and to avoid direct contact with potentially toxic or inedible taxa. Where possible, complete fruiting bodies were collected by carefully removing the stem from the upper topsoil or substrate. Each specimen was placed in an individually labelled sealed polypropylene bag, transported to the laboratory and processed separately.

Taxonomic identification was based initially on macroscopic characteristics and subsequently confirmed by DNA barcoding. Genomic DNA was extracted from approximately 100 mg of frozen homogenised mushroom material using the DNeasy Plant Mini Kit^®^ (Qiagen Inc., Germantown, MD, USA), following the manufacturer’s protocol. The internal transcribed spacer (ITS) region of fungal ribosomal DNA was amplified by polymerase chain reaction (PCR) using the ITS1 and ITS4 primers. Successful amplicons were purified and subjected to Sanger sequencing, and the resulting sequences were compared with reference sequences for taxonomic confirmation [42,43]. ITS amplification and sequencing were successful for 104 of the 121 reconstructed fruiting-body units (86.0%), all of which received a named taxonomic assignment. For the remaining 17 units (14.0%), genomic DNA was extracted, but repeated PCR attempts did not yield an ITS amplicon suitable for sequencing. These specimens could therefore not be submitted for Sanger sequencing, and insufficient residual material was available for further DNA extraction or assay optimisation. Unsuccessful PCR amplification despite repeated attempts has also been reported in fungal DNA-barcoding studies [44]; however, the specific cause was not determined experimentally in the present study. These 17 records were retained as “Unknown/unidentified taxon” for overall analytical summaries but were not assigned to any named taxon and were excluded from taxon-specific biological inference.

### 2.3. Mushroom Cleaning, Tissue Separation and Homogenisation

In the laboratory, visible debris, insects and adherent soil particles were manually removed from each fruiting body. Samples were washed with tap water, rinsed with deionised water and finally rinsed with Milli-Q ultrapure water produced using a Milli-Q^®^ Direct 8 system (Merck Millipore, Darmstadt, Germany; resistivity 18.2 MΩ·cm).

Where sufficient biomass was available, fruiting bodies were separated into cap and stem fractions before drying to allow tissue-specific evaluation. When biomass was limited, whole fruiting bodies were processed intact to prioritise taxon- and site-level analytical coverage. Cleaned mushroom material was oven-dried at 70–80 °C for 24 h, following procedures commonly used for elemental analysis of mushroom matrices [20,23]. Dried material was cryogenically homogenised with liquid nitrogen using a ceramic mortar and pestle to obtain a fine, visually uniform powder. The powdered material was passed through a 200-mesh sieve, thoroughly mixed and stored in pre-cleaned polyethylene bags or capped polypropylene tubes until digestion.

Reusable equipment, including forceps, spatulas, mortars, pestles and digestion vessels, was cleaned between samples using laboratory detergent, deionised water, 5% *v*/*v* nitric acid and repeated rinsing with Milli-Q ultrapure water. Glassware was avoided where possible for trace-metal work.

### 2.4. Fruiting-Body-Level Reconstruction

The original analytical dataset included whole fruiting bodies and tissue-separated cap/stem records. For the main manuscript, a reconstructed fruiting-body-level dataset was therefore generated. Whole fruiting bodies were retained one-to-one. When paired cap and stem records from the same fruiting body were available, the paired values were averaged without tissue-mass weighting as a pragmatic proxy for whole-fruiting-body composition.

This reconstruction was used to avoid inflating sample size through separate treatment of cap and stem subsamples from the same organism. It is not equivalent to mass-weighted reconstruction, because tissue masses were not available for all paired records, but it provides a transparent and conservative analytical unit for taxon-level and area-level summaries. The final main dataset comprised 121 reconstructed fruiting-body-level units. Concentrations are reported as ng g^−1^ dry weight (dw).

The original analytical-record dataset contained 157 mushroom records for the Ga–Ge–Nb–Ta–W panel, including tissue-level records. Four unpaired tissue-only records—one isolated cap record and three isolated stem records—could not be assigned to complete fruiting-body-level units and were excluded from reconstruction. The final reconstruction therefore used 153 analytical portions, comprising 89 whole-fruiting-body records and 32 paired cap–stem sets, to generate 121 fruiting-body-level units.

### 2.5. Mushroom Mineralisation and ICP-MS Determination

Dried and homogenised mushroom powders were mineralised using a closed-vessel acid digestion procedure in pre-cleaned Teflon digestion vessels. Where sufficient material was available, approximately 0.500 g of dry mushroom powder was accurately weighed into each vessel. Samples were digested using concentrated nitric acid and hydrogen peroxide, both Suprapur^®^ grade (Merck, Darmstadt, Germany), to oxidise the organic mushroom matrix. After digestion and cooling, digestates were filtered through Whatman™ Grade 42 ashless quantitative filter paper (Cytiva, Marlborough, MA, USA) and diluted to a final volume of 25 mL with Milli-Q ultrapure water.

Before ICP-MS analysis, an aliquot of each digest was passed through a 0.45 µm PTFE syringe filter to remove any residual fine particulate material and protect the sample-introduction system. Concentrations of Ga, Ge, Nb, Ta and W were determined by inductively coupled plasma mass spectrometry using a PerkinElmer NexION 2000B ICP-MS system (PerkinElmer, Waltham, MA, USA), operated with Syngistix Software for ICP-MS version 3.2 at the University of Oxford, Oxford, UK. Mushroom concentrations were converted to dry-weight values and reported as ng g^−1^ dry weight (dw).

### 2.6. Composite Topsoil Dataset

The topsoil dataset was used to characterise the broader geochemical setting of the monitored parks and green spaces. Surface topsoil was collected from the upper 0–5 cm horizon. The wider sampling programme comprised 850 field increments collected across 18 urban parks or open spaces and eight rural Leicestershire locations [42]. Within each location, the field increments were pooled and thoroughly homogenised to generate one site-level composite, resulting in 26 site-level composites. Each homogenised composite was subsequently processed in duplicate, producing 52 source records, corresponding to two records per site. Because paired records originated from the same site-level composite, they were not treated as independent environmental observations. For the present study, the two records were arithmetically averaged to obtain one value per monitored site; consequently, all topsoil summaries and contextual analyses were conducted using *N* = 26 site-level values. The 52 source records were retained only for analytical traceability and assessment of within-composite variability.

Duplicate subsamples were digested using microwave-assisted digestion with nitric acid and hydrochloric acid, both Suprapur^®^ grade, in a Multiwave GO microwave digestion system (Anton Paar GmbH, Graz, Austria). After cooling, digestates were filtered through Whatman™ Grade 42 ashless quantitative filter paper and diluted to 50 mL with Milli-Q ultrapure water. Before inductively coupled plasma mass spectrometry analysis, the mineralised samples were diluted in 5% *v*/*v* nitric acid and filtered through 0.45 µm syringe filters. Because the HNO_3_/HCl digestion did not include hydrofluoric acid and does not completely dissolve resistant silicate mineral phases, the resulting concentrations are described throughout as acid-recoverable or pseudo-total concentrations rather than total elemental concentrations. This limitation is particularly relevant to Nb and Ta, which may occur in resistant mineral phases [9,10,11,12]. The measured concentrations should therefore not be interpreted as complete soil elemental inventories or as estimates of labile or biologically available fractions.

### 2.7. Soil Physicochemical Characterisation

Soil physicochemical properties and texture were determined in homogenised composite topsoil material at IMIDRA using Spanish official soil analytical protocols [45]. These variables were retained as site-level contextual edaphic descriptors because pH, organic matter and texture can influence elemental retention, mobility and acid-recoverable concentrations in soil [46,47]. However, they were not used as predictors of individual mushroom concentrations because the composite topsoil samples were not collected beneath individual fruiting bodies or within their specific mycelial microhabitats. Assigning the same site-level topsoil value to multiple fruiting bodies would therefore introduce environmental pseudoreplication and imply a spatial correspondence not supported by the sampling design.

After air-drying and sieving through a 2 mm mesh, pH and electrical conductivity were measured in soil–water suspensions using a 1:2.5 *w*/*v* ratio of topsoil to deionised water. Briefly, 10 g of homogenised topsoil was mixed with 25 mL of deionised water and agitated for 20 min before measurement with calibrated probes. Moisture content was determined gravimetrically after drying at 105 °C to constant weight. Organic matter was determined using the Walkley–Black dichromate oxidation method. Soil texture was determined on the homogenised <2 mm fraction using the Bouyoucos hydrometer method.

### 2.8. Analytical Calibration, Verification and Quality Assurance

The mushroom ICP-MS method used peak-hopping acquisition with 20 sweeps per reading, one reading per replicate and five instrumental replicates. ^69^Ga, ^74^Ge and ^93^Nb were measured in helium kinetic-energy-discrimination (KED) mode, using helium flow rates of 5.0 mL min^−1^ for Ga and 3.9 mL min^−1^ for Ge and Nb. ^181^Ta and ^183^W were measured in standard mode without collision-cell gas. Dwell times were 25 ms for Ga and 20 ms for Ge, Nb, Ta and W, corresponding to integration times of 500 and 400 ms, respectively. The archived method applied a mathematical correction to the ^74^Ge signal based on ^77^Se (−0.116645 × ^77^Se); no mathematical interference correction was listed for the other four target isotopes. The corresponding topsoil exports recorded the same isotopes and cell-mode assignments. Numerical settings for radiofrequency (RF) power and argon plasma, auxiliary and nebuliser gas flows were not retained in the available archived exports and were therefore not reconstructed.

Analytical calibration was performed using a commercial multi-element calibration standard supplied by CPAchem Ltd. (Bogomilovo, Stara Zagora, Bulgaria), from which ten concentration levels were prepared for each target element. Calibration linearity was evaluated by unweighted ordinary least-squares regression with a freely estimated intercept, using nominal standard concentration as the predictor and net instrumental response as the dependent variable. An acceptance criterion of *R*^2^ > 0.995 was applied. All calibration curves for the target elements fulfilled this criterion in both analytical batches, and the corresponding *R*^2^ values are reported to six decimal places in Supplementary Table S1. Independently sourced commercial multi-element aqueous quality-control solutions supplied by QMx Laboratories Ltd. (Thaxted, Essex, UK) were included in the analytical batches to monitor instrumental performance independently of the CPAchem calibration standard. Their results were interpreted according to the calibration-range conditions described below.

Matrix reference materials were included in the broader multielement analytical programme. NIST SRM 1570a Trace Elements in Spinach Leaves (National Institute of Standards and Technology, Gaithersburg, MD, USA) was processed with the mushroom samples, whereas CRM059-50G Trace Metals–Loamy Clay 2, lot LRAA8361 (RTC, Laramie, WY, USA), was processed with the composite-topsoil samples. For the certified or reference elements evaluated in the broader analytical programme, previously reported recoveries ranged from 79.8% to 120.2% for NIST SRM 1570a and from 94.9% to 108.6% for CRM059-50G, supporting the digestion and analytical workflow for the metal(loid)s represented by assigned values in these materials [33]. However, neither NIST SRM 1570a nor CRM059-50G provided assigned mass-fraction values for Ga, Ge, Nb, Ta or W. Consequently, these two matrix reference materials could not be used to calculate element-specific recoveries for the target panel investigated in the present study.

An additional soil certified reference material, NCS DC87101 (China National Analysis Center for Iron and Steel, Beijing, China), was analysed in the topsoil sequence. Its certificate provides assigned values for Ga, Nb and W, but not for Ge or Ta. Retrospective evaluation of the archived target-element result did not demonstrate acceptable agreement with the assigned values. The NCS DC87101 result was therefore not used as evidence of target-element recovery or as a formal analytical acceptance criterion. Accordingly, although the broader matrix-reference-material results supported the digestion and measurement of the certified metal(loid)s evaluated previously, complete matrix-specific recovery validation is not claimed for the Ga–Ge–Nb–Ta–W panel. In this context, the concentrations reported for Ga, Ge, Nb, Ta and W should be interpreted as a blank-corrected environmental-occurrence baseline supported by calibration linearity, instrumental repeatability, digestion-batch blank correction and detection-limit procedures, but with incomplete target-element-specific, matrix-based accuracy and recovery validation.

Quality control during mushroom digestion included 18 acid-digestion blanks distributed across ten digestion batches. Before paired cap and stem measurements were combined at fruiting-body level, each analytical portion was corrected using the acid-digestion blank from the corresponding batch. When two blanks were included in the same batch, their arithmetic mean was used. For each element, the blank-corrected digestate concentration was calculated as:

$$C_{\mathrm{corrected},i,e}=\max\left[C_{\mathrm{sample},i,e}-C_{\mathrm{blank},b\left(i\right),e},\;0\right]$$

where $C_{\mathrm{sample},i,e}$ is the measured concentration of element e in analytical portion i, and $C_{\mathrm{blank},b\left(i\right),e}$ is the concentration of the same element in the acid-digestion blank, or the mean of the two blanks, included in the corresponding digestion batch. Negative sample-minus-blank differences were set to zero for non-negative concentration reporting. Blank-corrected digestate concentrations were converted to ng g^−1^ dry weight using the dry mass of each analytical portion and a final digest volume of 25 mL. On the original digestate-solution scale, the mean ± SD acid-blank concentrations used for blank correction and estimation of empirical variability were 0.183 ± 0.206 µg L^−1^ for Ga, 0.00321 ± 0.0136 µg L^−1^ for Ge, 0.307 ± 0.0216 µg L^−1^ for Nb, 5.52 ± 0.0230 µg L^−1^ for Ta and 1.49 ± 0.0612 µg L^−1^ for W. Five Ga blank results and 17 Ge blank results were reported below the instrumental detection limit; their original censored status was retained.

The instrumental detection limit (IDL) was calculated as three times the standard deviation of four initial 2% HNO_3_ wash measurements. For each analytical portion, the working limit of detection (LoD) was determined from the greater of the scale-adjusted IDL and three times the standard deviation of the 18 acid-digestion blanks, followed by conversion to dry-weight concentration using the corresponding sample mass. Results below the applicable LoD were treated as left-censored. Because 17 of the 18 Ge blank measurements were below the IDL, Ge detection was classified conservatively by requiring the uncorrected digestate concentration to exceed the highest quantified Ge blank. Classification based on the batch-specific blank correction was retained as a sensitivity analysis. Full blank and detection-limit information is provided in Supplementary Table S1.

Following digestion-batch blank correction, the mushroom working LoDs were analytical-portion-specific because conversion to dry-weight concentrations incorporated the individual mass of each analytical portion. The median and range of the resulting working LoDs for each element are reported in Supplementary Table S1. The aqueous QCA and QCB solutions were each measured on three occasions following automated twofold dilution. Because the nominal concentrations introduced into the ICP–MS exceeded the corresponding upper calibration limits, the resulting mean concentrations and relative standard deviations were used solely to characterise instrumental repeatability under extrapolated conditions. They were not interpreted as evidence of within-range accuracy or analytical recovery, and recovery percentages were therefore not calculated.

For topsoil digestates, the working LoDs were 0.01296 mg kg^−1^ dw for Ga, 0.01130 mg kg^−1^ dw for Ge, 0.02264 mg kg^−1^ dw for Nb, 0.03377 mg kg^−1^ dw for Ta and 0.31156 mg kg^−1^ dw for W. The complete calibration ranges, *R*^2^ values, instrumental and working detection limits, detection profiles and aqueous QCA/QCB repeatability results for both analytical matrices are provided in Supplementary Table S1.

### 2.9. Data Treatment and Statistical Analysis

At fruiting-body level, each element-specific result was classified as fully quantified, partially censored or fully censored. For fruiting bodies represented by paired cap and stem measurements, a result was classified as fully quantified when both components met the applicable detection criterion, partially censored when only one component was quantified and fully censored when neither component was quantified. The number of fruiting bodies with at least one quantified component was reported separately and was not treated as equivalent to the number fully quantified. For the present dataset, the ≥80% fully quantified criterion was applied at the overall analyte level as a deliberately conservative, study-specific rule for deciding whether an element would support continuous distributional interpretation across the complete 121-unit dataset. This rule was adopted to limit the influence of censoring and associated model assumptions on the principal continuous summaries [40,41] and should not be interpreted as a universally established statistical threshold or as a subgroup-specific cutoff. Ga was the only element meeting this overall criterion, with 106/121 (87.6%) fruiting-body-level units fully quantified.

Ga distributions were therefore summarised using regression on order statistics (ROS). At the overall fruiting-body level, ROS incorporated all 121 reconstructed units: the 106 fully quantified concentrations were entered as observed values, whereas the 15 fully censored observations were entered as left-censored observations with their applicable sample-specific censoring bounds. Helsel–Cohn plotting positions were used, with modelling on the logarithmic scale [40,41]. Modelled censored values were back-transformed and combined with the observed quantified values to obtain the mean, standard deviation, median and interquartile range. The same ROS approach was used descriptively for predefined Ga subgroups containing censored observations; the ≥80% criterion was not applied as a subgroup-specific cutoff, and subgroup estimates were interpreted cautiously in light of censoring, sample size and sampling imbalance. Fixed substitution using the LoD was not applied. P95 and P97.5 were not reported because their estimation was not considered sufficiently robust in the presence of censoring and small taxon-specific sample sizes. For groups without censored Ga observations, descriptive statistics were calculated directly from the observed concentrations.

For Ge, Nb, Ta and W, the high proportions of fully censored fruiting-body-level observations precluded robust estimation of continuous distributional summaries. These elements were therefore described using the numbers and percentages of fully quantified, partially censored and fully censored observations, together with the range of fully quantified concentrations where available. Cap–stem comparisons were restricted to pairs in which both tissues were quantified for the element under consideration. Taxon-level summaries were restricted to groups represented by at least five fruiting-body-level units, while spatial summaries were interpreted descriptively because of unequal sample sizes and differences in taxonomic composition.

For Ge, the primary and sensitivity classifications were used to evaluate the dependence of low-level occurrence classification on blank treatment. Because 17 of the 18 Ge acid-digestion blank measurements were below the instrumental detection limit, the primary analysis used the conservative maximum-blank criterion described in Section 2.8, whereas the sensitivity analysis reclassified the same analytical records using digestion-date-specific blank correction. The sensitivity analysis was used solely to assess the robustness of the Ge censoring classification to the blank-treatment approach; continuous Ge distributional estimates were not calculated under either criterion.

For continuous multielement ordination, at least three elements were required to satisfy the same study-specific quantitative-coverage criterion. Following blank correction and application of the sample-specific detection criteria, only Ga met this requirement. Principal component analysis and continuous mushroom inter-element correlation analysis were therefore not performed, as multivariate analysis of the remaining elements would have been dominated by censoring and detection-limit structure.

Site-level topsoil physicochemical variables were summarised descriptively using the median, interquartile range and observed range for the 26 monitored locations. Cross-matrix correlations between these site-level descriptors and individual mushroom concentrations were not calculated because the two matrices were not paired at fruiting-body or mycelial-microhabitat scale. Repeating a composite topsoil value across multiple fruiting bodies would have inflated the effective sample size, whereas aggregation of mushroom concentrations by collection location would have produced a small and highly unbalanced dataset confounded by taxonomic composition and analytical censoring.

Supplementary contextual topsoil geochemical screening calculations were performed only to support the geochemical interpretation of the composite topsoil dataset. Pollution index (PI) and Fe-normalised enrichment factor (EF) values were calculated for Ga, Ge, Nb, Ta and W using national median reference values from the *Advanced Soil Geochemical Atlas of England and Wales* [48]. The Atlas median values used were 10 mg kg^−1^ for Ga, 0.93 mg kg^−1^ for Ge, 12 mg kg^−1^ for Nb, 0.70 mg kg^−1^ for Ta, 2.1 mg kg^−1^ for W and 2.8% Fe, converted to 28,000 mg kg^−1^ where required for unit consistency. PI was calculated as C_element,sample_/C_element,Atlas_ median, whereas EF was calculated as (C_element_/Fe)_sample_/(C_element_/Fe)_Atlas_ median. Iron (Fe) concentrations used for the enrichment-factor calculations were measured in the same 26 composite topsoil samples and were obtained from the dataset reported in the doctoral thesis [33]. Because the concentrations generated in the present study represent acid-recoverable rather than complete total concentrations, and because the Atlas medians were generated within a different national-scale sampling and analytical framework, the PI and EF values were treated only as comparative geochemical screening ratios. They were not interpreted as formal pollution or enrichment classifications, regulatory exceedances, source-apportionment evidence, estimates of bioavailability or evidence of soil-to-fungus transfer. Site-level PI and EF values for the 26 monitored locations are reported in Supplementary Table S2, while the Fe-normalised EF distributions are displayed graphically in Figure S1. Because the study concentrations and Atlas reference values were generated using methodologically non-equivalent analytical frameworks, these ratios were used only for comparative environmental screening.

## 3. Results

### 3.1. Analytical Performance and Detection Profile

All calibration curves fulfilled the acceptance criterion of *R*^2^ > 0.995 in both analytical batches, with the corresponding values reported to six decimal places in Supplementary Table S1. Repeated QCA/QCB measurements produced RSDs of 1.86–6.16% for the mushroom batch and 1.35–5.87% for the topsoil batch. Because the nominal concentrations introduced into the ICP–MS exceeded the corresponding upper calibration limits, these results were interpreted solely as measures of instrumental repeatability under extrapolated conditions and not as evidence of within-range accuracy or analytical recovery. Recovery percentages were therefore not calculated.

**Figure 2:**
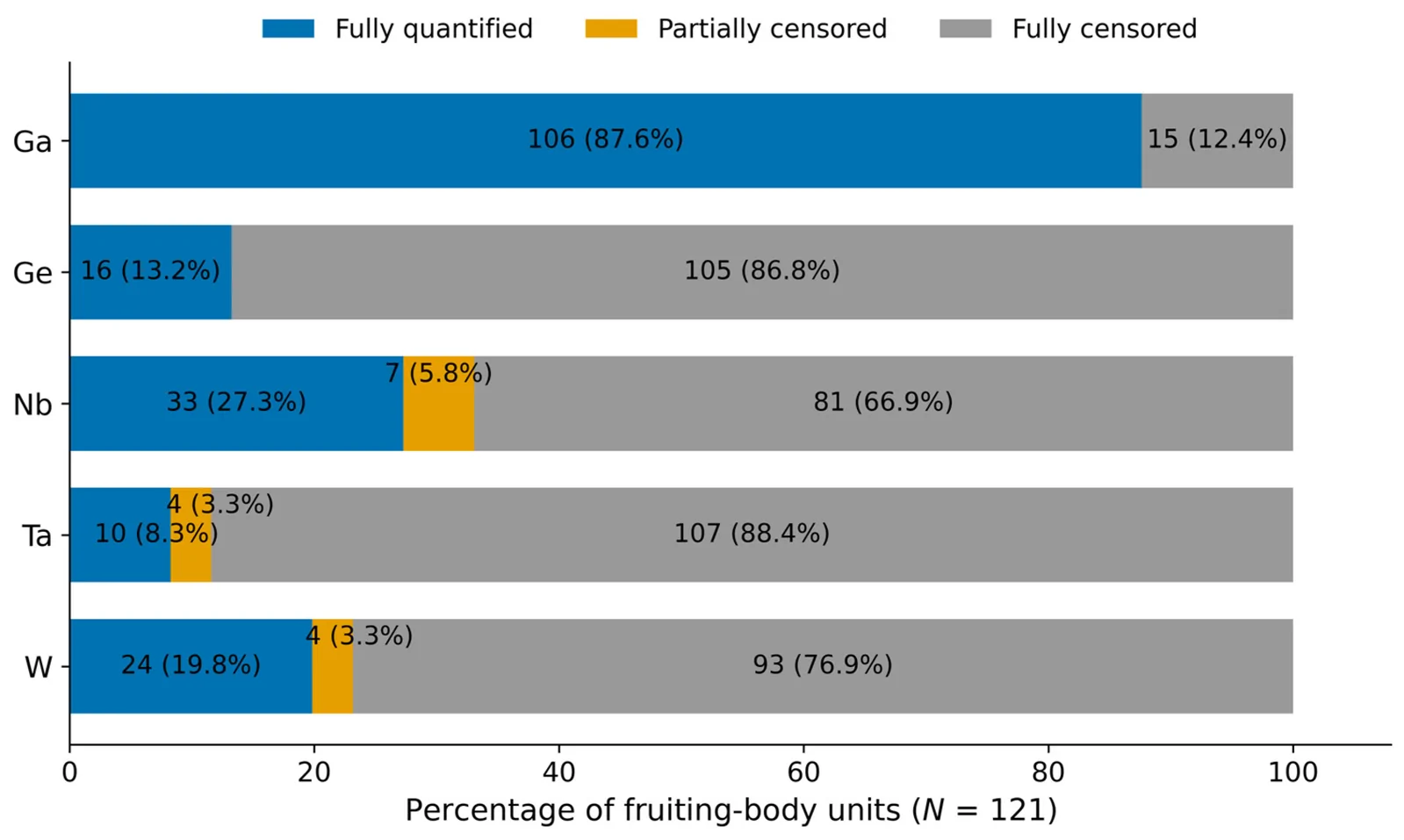
Blank-corrected censoring profiles for Ga, Ge, Nb, Ta and W in 121 reconstructed wild-mushroom fruiting-body-level units. Bars show the percentages classified as fully quantified (FQ), partially censored (PC) and fully censored (FC), and labels report *n* (%). For fruiting bodies represented by paired cap and stem measurements, FQ indicates that both components met the applicable detection criterion, PC indicates that only one component was quantified, and FC indicates that neither component was quantified. Ge classifications use the conservative maximum-blank criterion selected for the primary analysis. Percentages may not sum exactly to 100 because of rounding.

The blank-corrected reconstructed dataset comprised 121 fruiting-body-level units (Table 1; Figure 2). Ga was fully quantified in 106 units (87.6%) and fully censored in 15 (12.4%); no units were partially censored. Under the conservative primary criterion for Ge, 16 units (13.2%) were fully quantified and 105 (86.8%) were fully censored. The date-specific blank-correction sensitivity criterion classified 24 units as having at least one quantified component—21 fully quantified and three partially censored—but neither Ge classification was interpreted as a robust estimate of environmental prevalence. For Nb, 33 units were fully quantified, seven partially censored and 81 fully censored; the corresponding classifications were 10, four and 107 for Ta and 24, four and 93 for W. Consequently, Ga was the only element with sufficient quantitative coverage for distributional estimation, whereas Ge, Nb, Ta and W were summarised primarily through censoring classifications and the ranges of fully quantified observations.

### 3.2. Overall Fruiting-Body-Level Concentrations

Following blank correction, Ga was the only element for which a distributional summary was considered sufficiently supported (Table 1). Regression on order statistics yielded a median Ga concentration of 475.48 ng g^−1^ dw and an interquartile range of 278.70–941.63 ng g^−1^ dw. The 106 fully quantified Ga observations ranged from 25.51 to 14,410.84 ng g^−1^ dw, while the remaining 15 units were fully censored at their applicable sample-specific limits. The broad quantified range and the difference between the median and the upper quantified values indicate a strongly right-skewed distribution.

For Ge, Nb, Ta and W, the extent of censoring precluded defensible estimation of medians, interquartile ranges or upper percentiles. Their fully quantified observations ranged from 3.36 to 45.99 ng g^−1^ dw for Ge, 3.49 to 256.49 ng g^−1^ dw for Nb, 4.58 to 50.91 ng g^−1^ dw for Ta and 9.79 to 1160.68 ng g^−1^ dw for W. These ranges describe only the fully quantified subset and should not be interpreted as estimates of the complete fruiting-body distributions. Partially censored units for Nb, Ta and W were retained as a separate analytical category and were not incorporated into continuous distributional summaries.

### 3.3. Taxon-Level Profiles

**Figure 3:**
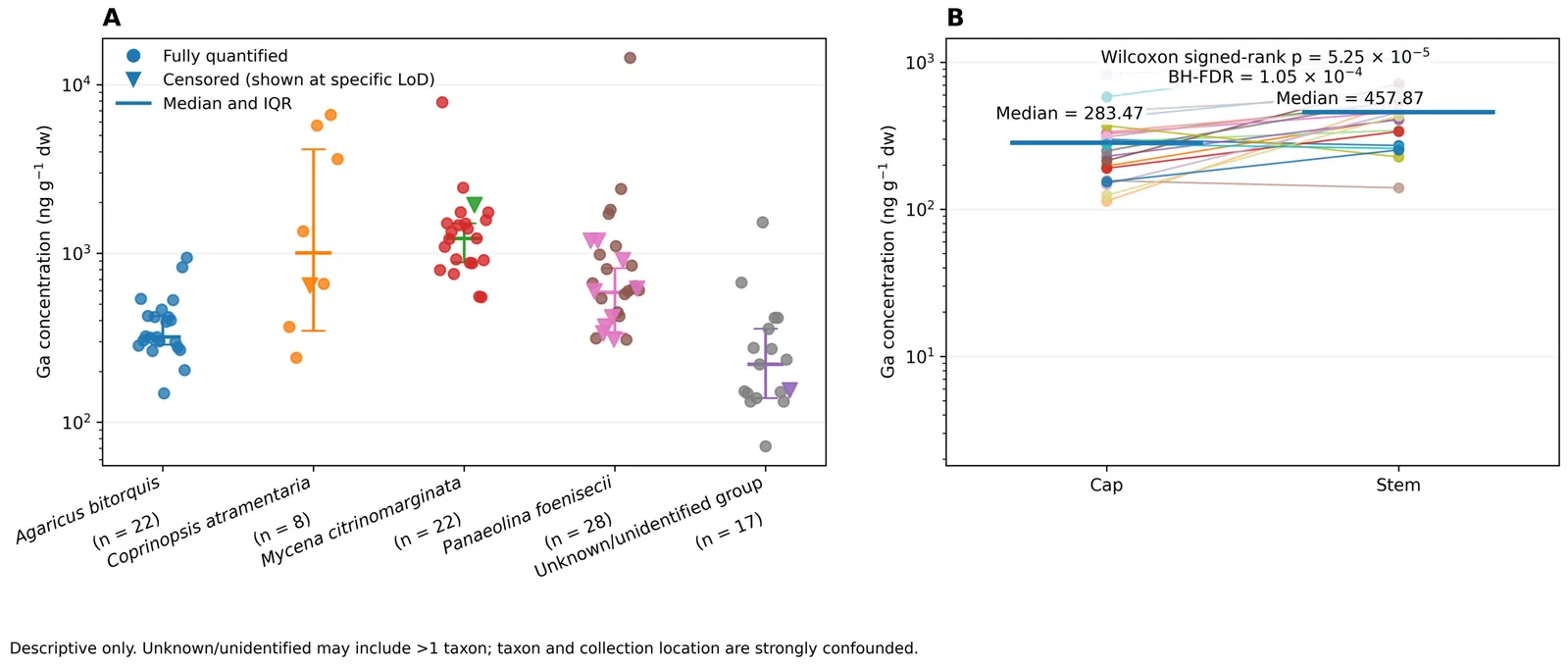
Blank-corrected Ga patterns in wild-mushroom fruiting bodies. (**A**) Ga concentrations in the five principal analytical groups. Circles represent fully quantified fruiting-body-level observations, whereas downward triangles represent censored observations displayed at their corresponding sample-specific limits of detection (LoDs). Horizontal lines and error bars show the median and interquartile range, respectively. Colours are used for visual separation of the plotted observations and do not encode an additional analytical variable; quantification status is indicated by symbol shape. Taxon-level patterns are descriptive because taxon and collection location were strongly confounded, and the unknown/unidentified group may contain more than one taxon. (**B**) Paired cap–stem Ga concentrations in 22 *Agaricus bitorquis* fruiting bodies. Lines connect tissues from the same fruiting body. Different line colours are used only to facilitate visual tracking of individual paired fruiting bodies and do not denote analytical or biological groups. The Wilcoxon signed-rank *p*-value and Benjamini–Hochberg false-discovery-rate-adjusted *p*-value are shown. Concentrations are expressed as ng g^−1^ dry weight (dw), and both panels use logarithmic y-axes.

Taxon-level results are presented in Table 2 for the five analytical groups represented by at least five reconstructed fruiting-body-level units: *Agaricus bitorquis* (*n* = 22), *Coprinopsis atramentaria* (*n* = 8), *Mycena citrinomarginata* (*n* = 22), *Panaeolina foenisecii* (*n* = 28) and the unknown/unidentified analytical group (*n* = 17). Table 2 reports the censoring classifications and fully quantified ranges for all five elements, whereas distributional summaries were restricted to Ga because Ge, Nb, Ta and W did not retain sufficient quantitative coverage within individual groups. The blank-corrected Ga distributions are presented in Figure 3A. Taxa represented by fewer than five units are reported separately in Supplementary Table S3. The principal analytical groups were not distributed uniformly across the monitoring locations. In particular, all *C. atramentaria* units originated from Bradgate Park, whereas all units in the Unknown/unidentified analytical group originated from Castle Gardens. Taxon-level patterns were therefore interpreted descriptively because fungal identity and location could not be separated statistically in several groups.

Ga was fully quantified in all 22 *A. bitorquis* units, seven of the eight *C. atramentaria* units, 21 of the 22 *M. citrinomarginata* units, 19 of the 28 *P. foenisecii* units and 16 of the 17 unknown/unidentified units. The observed median for *A. bitorquis* was 320.60 ng g^−1^ dw, with an IQR of 288.57–424.28 ng g^−1^ dw. For groups containing censored observations, regression-on-order-statistics medians were 1004.78 ng g^−1^ dw for *C. atramentaria*, 1223.45 ng g^−1^ dw for *M. citrinomarginata*, 585.58 ng g^−1^ dw for *P. foenisecii* and 220.56 ng g^−1^ dw for the unknown/unidentified group. The corresponding IQRs were 347.38–4143.68, 886.72–1505.27, 335.35–817.26 and 138.92–357.56 ng g^−1^ dw, respectively.

Quantitative coverage for the other elements remained sparse within the four named taxa. Depending on the taxon, the numbers of fully quantified observations ranged from zero to two for Ge, zero to four for Nb, zero to one for Ta and one to two for W. The unknown/unidentified analytical group contained six fully quantified observations for Ge, 11 for Nb, nine for Ta and 14 for W, but these values were not used for biological inference because the group may contain multiple taxa. All taxon-level patterns are descriptive because sampling was unbalanced and taxon was strongly confounded with collection location. The results for *C. atramentaria* are particularly tentative because *n* = 8 and its fully quantified Ga concentrations ranged from 241.27 to 6622.19 ng g^−1^ dw.

All 17 units in the unknown/unidentified analytical group were collected at Castle Gardens on 12 September 2019. Under the conservative primary Ge criterion, six units were fully quantified and 11 were fully censored. No named taxon was collected at this location within the present dataset. Taxonomic identity, collection location and collection date are therefore completely confounded for this analytical group, and its apparently greater Ge detectability cannot be attributed to fungal taxonomy.

### 3.4. Tissue-Specific Patterns in Agaricus bitorquis

*Agaricus bitorquis* was the only taxon with sufficient paired cap–stem replication for a main-text tissue-specific assessment. The analysis used the original blank-corrected tissue-level records rather than the reconstructed fruiting-body-level averages. Following application of the analytical-portion-specific detection criteria, Ga was fully quantified in all 22 paired fruiting bodies. Median Ga concentrations were 283.47 ng g^−1^ dw in caps and 457.87 ng g^−1^ dw in stems, with corresponding IQRs of 191.80–333.68 and 340.12–550.20 ng g^−1^ dw, respectively. Ga concentrations were significantly higher in stems than in the corresponding caps (Wilcoxon signed-rank *p* = 5.25 × 10^−5^; BH-FDR = 1.05 × 10^−4^; Table 3; Figure 3B).

Quantitative coverage was insufficient for comparable tissue-specific interpretation of Ge, Nb, Ta and W. All 22 Ge pairs were fully censored. For Nb, three pairs were fully quantified, five were partially censored and 14 were fully censored; the corresponding classifications were 0, 4 and 18 pairs for Ta and 2, 2 and 18 pairs for W. Consequently, no continuous tissue-specific summaries or paired inferential tests were calculated for these four elements, and no conclusions regarding their cap–stem partitioning were drawn.

### 3.5. Area-Type and Urban-Quadrant Descriptive Patterns

Area-type results are presented in Supplementary Table S4. The dataset comprised 113 urban and eight rural fruiting-body-level units. The rural subset was restricted to eight *Coprinopsis atramentaria* units collected at Bradgate Park, whereas the urban subset included 17 named or unidentified analytical groups collected across 12 locations. Area type, fungal taxonomy and collection location were therefore strongly confounded, and the following results should not be interpreted as a controlled urban–rural comparison or as a county-wide rural baseline.

Ga was fully quantified in 99/113 urban units and 7/8 rural units. Regression on order statistics yielded a median Ga concentration of 464.36 ng g^−1^ dw in the urban subset, with an IQR of 276.32–910.69 ng g^−1^ dw. The corresponding rural median was 1004.78 ng g^−1^ dw, with an IQR of 347.38–4143.68 ng g^−1^ dw. Fully quantified Ga concentrations ranged from 25.51 to 14,410.84 ng g^−1^ dw in urban units and from 241.27 to 6622.19 ng g^−1^ dw in rural units. The broader and higher rural distribution largely reflects the eight *C. atramentaria* fruiting bodies represented in this subset and cannot be attributed independently to rural environmental conditions.

Quantitative coverage for Ge, Nb, Ta and W remained insufficient for continuous area-type summaries. In the urban subset, Ge was fully quantified in 14 units; Nb was fully quantified in 29 and partially censored in seven; Ta was fully quantified in 10 and partially censored in four; and W was fully quantified in 23 and partially censored in four. In the rural subset, Ge and Nb were fully quantified in two and four units, respectively, whereas Ta was fully censored in all eight units and W was fully quantified in only one. These results describe element-specific occurrence within the sampled subsets but do not support comparisons of central tendency between urban and rural mushrooms.

Urban-quadrant results are presented in Supplementary Table S5. The NE, NW, SE and SW subsets comprised 30, 51, 27 and five fruiting-body-level units, respectively. Ga was fully quantified in 21/30 NE, 49/51 NW, 25/27 SE and 4/5 SW units. ROS-based median Ga concentrations were 565.66 ng g^−1^ dw in NE, 322.38 ng g^−1^ dw in NW, 922.92 ng g^−1^ dw in SE and 572.96 ng g^−1^ dw in SW. The corresponding IQRs were 253.32–837.99, 249.90–511.67, 768.85–1486.33 and 505.86–598.21 ng g^−1^ dw, respectively. The SW estimate is particularly uncertain because it was based on only five units.

The apparent quadrant differences cannot be separated from taxonomic and site composition. The NE subset was dominated by *Panaeolina foenisecii* (23/30 units), the NW subset principally comprised *Agaricus bitorquis* (22/51) and the unknown/unidentified analytical group (17/51), the SE subset was dominated by *Mycena citrinomarginata* (22/27), and all five SW units were *P. foenisecii* collected at Braunstone Park. The quadrant-specific censoring profiles for Ge, Nb, Ta and W were also heterogeneous, but the numbers of fully quantified observations were insufficient for defensible continuous summaries. In particular, the SW subset contained no fully quantified Ge, Nb or Ta observations, while its W result was based on a single fully quantified value.

Overall, the area-type and urban-quadrant results demonstrate heterogeneity in Ga concentrations and in the occurrence profiles of the other four elements. However, unequal sample sizes, extensive censoring, taxonomic imbalance and the concentration of several analytical groups within individual locations prevent attribution of these patterns to controlled spatial gradients. These descriptive outputs are therefore most appropriately used to identify priorities for future balanced, taxon-specific and spatially replicated sampling.

### 3.6. Composite Topsoil Concentrations and Environmental Framing

The composite topsoil dataset showed concentrations above the corresponding working LoDs for all five elements in each of the 26 site-level values obtained by averaging the two source records corresponding to each monitored site (Table 4).

The composite topsoil dataset showed concentrations above the corresponding working LoDs for all five elements in each of the 26 site-level values obtained by averaging the two source records corresponding to each monitored site. Median acid-recoverable concentrations were 59.84 mg kg^−1^ dw for Ga, 1.367 mg kg^−1^ dw for Ge, 0.860 mg kg^−1^ dw for Nb, 1.0108 mg kg^−1^ dw for Ta and 0.704 mg kg^−1^ dw for W. The corresponding IQRs were 50.83–70.99, 1.292–1.488, 0.839–0.908, 1.0095–1.0117 and 0.692–0.722 mg kg^−1^ dw, respectively. These site-level values describe the acid-recoverable geochemical composition of the monitored parks and green spaces and provide contextual environmental information for the mushroom dataset; they do not represent total soil elemental inventories or mushroom-specific substrate concentrations.

The site-level composite topsoil Ge concentration at Castle Gardens was 1.697 mg kg^−1^ dw, the second-highest of the 26 monitored locations. However, this park-scale composite was not collected directly beneath the unknown/unidentified fruiting bodies and does not represent their specific substrate or mycelial microhabitat. It is therefore reported only as contextual information and cannot establish the Ge concentration or bioavailable fraction to which those fruiting bodies were exposed.

The 26 site-level composite topsoil values had a median pH of 6.56 (IQR: 6.02–6.98) and a median electrical conductivity of 0.355 dS m^−1^ (IQR: 0.279–0.452 dS m^−1^). Median organic matter and moisture contents were 11.09% (IQR: 8.71–13.40%) and 3.58% (IQR: 3.04–4.31%), respectively. Median clay, sand and silt contents were 22.3% (IQR: 19.1–28.5%), 24.5% (IQR: 18.0–29.4%) and 52.3% (IQR: 48.1–54.3%), respectively. These measurements characterise the monitored topsoils at site-composite scale and were not interpreted as mushroom-specific substrate properties.

Site-level comparative geochemical screening ratios based on PI and Fe-normalised EF are reported in Supplementary Table S2. Figure S1 provides a graphical representation of the Fe-normalised EF distributions, calculated using Fe concentrations measured in the same 26 composite topsoil samples and reported in the doctoral thesis [33], together with national median reference values from the *Advanced Soil Geochemical Atlas of England and Wales* [48]. Figure S2 shows the correlation structure among Ga, Ge, Nb, Ta and W in the 26 site-level topsoil values. Because the corresponding continuous mushroom inter-element correlation analysis was removed following the censoring-aware reanalysis, the topsoil correlation structure was not contrasted with fungal inter-element covariation and was not interpreted as evidence of selective accumulation, passive reflection of soil composition or soil-to-fungus transfer. The study concentrations represent HNO_3_/HCl acid-recoverable fractions, whereas the Atlas medians were generated within a different national-scale sampling and analytical framework. The study and reference values are therefore not method-equivalent. Accordingly, the PI and EF values are interpreted only as comparative geochemical screening ratios and not as formal measures of pollution, enrichment, regulatory exceedance, bioavailability or source attribution. None of these topsoil-only outputs was used to infer individual mushroom uptake or soil-to-fungus transfer.

The soil and mushroom datasets operate at different but complementary scales. Topsoil composites summarise park-level geochemistry, whereas mushroom concentrations reflect fruiting-body-level biological, taxonomic and microhabitat influences. This scale difference is important when interpreting apparent soil–mushroom patterns, particularly for Nb and Ta, which are often strongly mineral-hosted, and for W, whose mobility depends on tungstate chemistry, pH, organic matter and microbial interactions [13,14,16,17,18,19].

## 4. Discussion

### 4.1. Baseline Occurrence, Analytical Censoring and Toxicological Relevance of Ga, Ge, Nb, Ta and W in Wild Mushrooms

This study provides baseline occurrence data for Ga, Ge, Nb, Ta and W in wild mushrooms collected from Leicester and Leicestershire. Following digestion-batch blank correction and application of the sample-specific detection criteria, Ga was the only element with sufficient quantitative coverage for continuous distributional estimation, being fully quantified in 106 of the 121 reconstructed fruiting-body-level units. Ge, Nb, Ta and W were affected by substantially greater censoring and were therefore interpreted using explicit censoring classifications and the ranges of fully quantified observations rather than modelled central distributions. The findings consequently document analytical occurrence within the sampled fruiting bodies, but do not demonstrate uniform detection, contamination, source attribution or element-specific accumulation capacity.

These results neither demonstrate a food-safety concern nor establish the absence of toxicological risk. Risk characterisation for technology-critical elements in wild edible mushrooms is constrained by limitations in the exposure, hazard and analytical evidence available. Exposure could not be estimated because the sampling programme was designed to investigate environmental occurrence rather than mushrooms entering the food chain. The dataset does not provide representative consumption frequencies or portion sizes, fresh-weight concentrations, the effects of washing or culinary processing, gastrointestinal bioaccessibility or element-specific chemical forms. Total dry-weight concentrations cannot therefore be translated directly into an ingested, absorbed or biologically effective dose.

Hazard characterisation is also uncertain because food-specific health-based guidance values, chronic oral dose–response relationships and toxicokinetic information are comparatively limited for Ga, Ge, Nb, Ta and W relative to more extensively studied foodborne elemental contaminants [1,3,13]. The possible influence of chemical speciation, repeated low-dose exposure, susceptible consumer groups and combined exposure to multiple technology-critical elements remains insufficiently characterised. Analytical censoring introduces an additional limitation: only Ga supported continuous distributional estimation, whereas the concentration distributions of Ge, Nb, Ta and W could not be robustly reconstructed. Consequently, calculation of estimated dietary intakes, hazard quotients or cumulative hazard indices would have been dominated by unsupported exposure assumptions, uncertain toxicological reference values and detection-limit structure. Nevertheless, the present occurrence data remain toxicologically relevant because they identify Ga as the principal quantitatively tractable endpoint and define the analytical and biological evidence required for future targeted risk assessment in edible wild-mushroom taxa.

The sparse literature for Ga, Ge, Nb, Ta and W in mushrooms makes direct comparison difficult. Previous multielement mushroom studies have shown that wild fungi can accumulate a wide range of trace elements, rare earth elements and platinum-group elements, but most fungal monitoring remains focused on classical metal(loid)s such as Cd, Pb, Hg, As, Cu, Zn, Mn and Ni [20,23,26,27,28]. Nevertheless, selected studies provide useful context. Mleczek et al. [27] reported Ga and Ge in wild mushrooms collected near a heavily trafficked road, while Falandysz et al. [29] reported low-level Ga, Ge, Nb, Ta and W in *Macrolepiota procera*. Other studies have reported Ga in selected fungal species such as *Amanita muscaria*, *Leccinum scabrum* and *Cordyceps kyushuensis* [30,31,49]. The present dataset therefore fills an important descriptive gap by providing a focused Ga–Ge–Nb–Ta–W panel in field-collected wild mushrooms using a fruiting-body-level analytical unit and explicit censoring-aware interpretation.

### 4.2. Ge as a Highly Censored Endpoint

Ge showed the greatest sensitivity to the treatment of acid-digestion blanks and analytical censoring. Under the conservative maximum-blank criterion selected for the primary analysis, only 16 of the 121 fruiting-body-level units were fully quantified, while 105 were fully censored, corresponding to 86.8% censoring. Under the alternative date-specific blank-correction criterion, 21 units were fully quantified, three were partially censored and 97 were fully censored; thus, 24/121 units contained at least one quantified component. The difference between these classifications demonstrates that the apparent occurrence frequency of Ge is sensitive to the treatment of low-level measurements and blank variability. Neither result should therefore be interpreted as a robust estimate of environmental prevalence. A median, IQR or upper percentile was not estimated for Ge.

This analytical uncertainty is consistent with the broader environmental literature, which describes Ge as a comparatively under-studied element that frequently occurs at low concentrations and whose behaviour depends strongly on matrix composition, speciation and geochemical conditions [3,7]. The available fungal literature is also limited and has addressed Ge primarily through multielement occurrence studies or controlled substrate-supplementation experiments rather than systematic monitoring of naturally occurring wild mushrooms [27,29,32]. Methodological caution in the present study therefore means that Ge is treated as an exploratory occurrence endpoint rather than as a continuously quantified variable. Interpretation is restricted to the censoring classifications, the range of fully quantified observations and comparison of the conservative primary and date-specific sensitivity criteria. The data do not support robust comparisons of Ge central tendency among taxa, locations, area types or tissues. Future studies should seek lower sample-specific detection limits, larger and more balanced taxon-specific datasets, replicated sampling across locations and targeted collection from settings where Ge availability may be higher. Statistical treatment should preserve the distinction between quantified and censored observations and apply censoring-aware methods when sufficient quantitative coverage is available, rather than substituting a fixed fraction of the detection limit.

The apparently greater Ge detectability in the unknown/unidentified analytical group cannot be separated from site context. All 17 units in this group were collected at Castle Gardens during the same sampling event, and six were fully quantified under the conservative primary criterion. The Castle Gardens site-level composite topsoil also contained 1.697 mg kg^−1^ dw Ge, the second-highest concentration among the 26 monitored locations. However, no named taxon was collected at Castle Gardens within this dataset, and the composite topsoil was not sampled directly beneath the fruiting bodies or within their corresponding mycelial microhabitats. Taxonomic identity, location and sampling event are therefore completely confounded. The observed pattern could reflect site context, substrate heterogeneity, sampling structure or other unmeasured factors and cannot be interpreted as evidence of taxon-specific Ge accumulation or direct soil-to-fungus transfer.

### 4.3. Taxon- and Tissue-Level Interpretation

Ga was the only element with sufficient quantitative coverage for continuous taxon-level interpretation. Its distributions varied descriptively among the five principal analytical groups, but these contrasts should not be interpreted as evidence of fixed taxon-specific accumulation capacity. Sampling was unbalanced, several taxa were concentrated within individual locations, and fungal identity could not be separated statistically from site and, in some cases, collection date. *Coprinopsis atramentaria* was represented by only eight fruiting-body-level units, all collected at Bradgate Park, whereas the 17 unknown/unidentified units were all collected at Castle Gardens. The corresponding group-level patterns therefore remain hypothesis-generating rather than inferential.

Nevertheless, taxon-aware interpretation remains important in fungal elemental studies. Fungal taxa can differ in trophic strategy, substrate use, mycelial distribution, elemental binding and transport processes, fruiting-body morphology and phenology [20,23,24,28,50]. Different taxa collected within the same broad environmental setting may therefore integrate distinct substrates and microhabitats. Consequently, pooled “wild mushroom” summaries can obscure biologically relevant heterogeneity. In the present study, however, the sampling structure does not allow the effects of fungal taxonomy to be separated from those of location, substrate and collection date.

For Ge, Nb, Ta and W, taxon-level information is restricted to the numbers of fully quantified, partially censored and fully censored observations and to the ranges of fully quantified values. The available quantitative coverage does not support comparisons of means, medians, upper tails or continuous multielement profiles among taxa. Restricting interpretation in this way avoids generating group-level differences driven primarily by substitution assumptions or detection-limit structure [40,41]. Future studies should use balanced taxon-specific replication across several locations and sampling dates to distinguish fungal biological variability from site, substrate and microhabitat effects.

Tissue-specific inference was likewise supported only for Ga. Previous fungal studies have shown that elemental partitioning between caps and stems may vary according to the element, fungal taxon and environmental conditions [22,24,39]. In the present dataset, *Agaricus bitorquis* was the only taxon with sufficient paired cap–stem replication, and Ga was fully quantified in all 22 pairs. Its higher concentration in stems provides evidence of differential within-fruiting-body distribution, although the data do not identify the uptake or translocation mechanism responsible. Ge, Nb, Ta and W lacked sufficient numbers of fully quantified pairs for continuous tissue comparisons; consequently, no conclusions regarding their cap–stem partitioning were drawn.

Reconstruction at fruiting-body level avoided treating matched cap and stem measurements as independent mushrooms while preserving the original tissue-level records for the supported paired analysis. The absence of complete tissue-mass data remains a limitation because an arithmetic mean of cap and stem concentrations is not equivalent to a mass-weighted whole-fruiting-body concentration. Nevertheless, unweighted reconstruction is more transparent than double-counting paired tissues. Future studies should record separate cap and stem dry masses to enable mass-weighted fruiting-body reconstruction.

### 4.4. Composite Topsoil Context and Scale Limitations

The composite topsoil dataset provides site-level geochemical context for the monitored parks and green spaces, but it does not represent the substrate immediately surrounding individual fruiting bodies or their corresponding mycelial microhabitats. The 26 composites summarise park-scale acid-recoverable topsoil chemistry, whereas the mushroom measurements represent fruiting-body-level biological profiles influenced by fungal taxonomy, trophic strategy, substrate use, tissue composition and local microenvironmental conditions. These complementary but non-equivalent spatial scales preclude calculation of defensible bioaccumulation or soil-to-fungus transfer factors. They also prevent attribution of individual mushroom concentrations to particular soil sources or measurements of bioavailability. Repeatedly assigning the same site-level composite value to multiple fruiting bodies would additionally create environmental pseudoreplication and overstate the effective sample size.

The contextual pollution index and Fe-normalised enrichment factor calculations require similar caution. The *Advanced Soil Geochemical Atlas of England and Wales* provides a useful national reference framework [48], but its medians are neither site-specific Leicester/Leicestershire background values nor method-equivalent to the HNO_3_/HCl acid-recoverable concentrations generated in the present study. The PI and EF outputs are therefore interpreted only as comparative geochemical screening ratios. They do not constitute formal classifications of pollution or enrichment, regulatory exceedances, estimates of bioavailability, evidence of source attribution or indicators that the measured topsoil pools were directly accessible to individual fruiting bodies.

This distinction is particularly relevant to the elements investigated. Nb and Ta are high-field-strength elements commonly associated with resistant mineral phases and generally low mobility under many environmental conditions, although biological uptake has been documented in particular environmental settings [9,10,11,12]. W mobility may vary more substantially because tungstate behaviour is influenced by pH, mineral and oxide surfaces, organic matter, microbial processes and chemical speciation [13,16,17,18,19]. Ga and Ge likewise show matrix- and speciation-dependent behaviour and may occur at low concentrations close to analytical detection limits [3,5,6,8]. Consequently, measurable acid-recoverable concentrations in composite topsoil cannot be assumed to represent the labile or biologically accessible pools encountered by fungal mycelia.

Future studies designed specifically to investigate soil-to-fungus transfer should collect fruiting bodies together with directly paired substrate soil, litter, mycelial-zone material and, where ecologically relevant, rhizosphere-associated soil at the same microhabitat scale. Soil porewater—the water occupying the spaces between soil particles and containing the operationally dissolved elemental fraction—should also be analysed to provide information closer to the pool potentially accessible to fungal mycelia than acid-recoverable bulk-soil concentrations. Porewater concentrations should nevertheless be interpreted alongside chemical speciation, dissolved organic matter, pH, redox conditions, Fe/Al/Mn oxide interactions, microbial ligands and sorption–desorption processes, because the dissolved fraction alone does not constitute a complete measure of biological availability [51]. Combining these measurements with repeated taxon-specific sampling would provide a substantially stronger basis for evaluating uptake pathways and element partitioning among soil, substrate, mycelium and fruiting-body tissues.

### 4.5. Environmental Monitoring Implications

Following digestion-batch blank correction and application of sample-specific detection criteria, Ga was the only element with sufficient quantitative coverage for continuous interpretation, whereas Ge, Nb, Ta and W were quantified only in subsets of the fruiting-body-level units. Within the sampled Leicester and Leicestershire settings, these findings suggest that wild mushrooms may have exploratory value as complementary matrices for environmental surveillance of selected technology-critical elements. This potential is site-, element- and taxon-dependent, requires censoring-aware interpretation and cannot be generalised beyond the present sampling design. Rather than functioning as universal bioindicators, wild mushrooms may provide complementary evidence of environmental occurrence when interpreted alongside substrate ecology, fungal taxonomy, local geochemical context and microhabitat-scale information [20,23,24,50]. This interpretation is consistent with the broader use of wild fungi in elemental monitoring, while recognising that fungal fruiting bodies integrate biological and environmental influences rather than passively reflecting bulk soil composition.

The environmental-monitoring relevance of these findings is reinforced by the current European critical-raw-materials context. The European critical raw materials framework identifies several technology-related elements as strategically important because of their economic relevance, supply vulnerability and role in high-technology applications [2,4]. Ga, Ge and W are identified as strategic raw materials, whereas Nb and Ta are included within the broader critical-raw-materials policy landscape [2,4]. This policy classification should not be interpreted as a toxicological ranking. Instead, it indicates that the industrial flows of these elements are likely to increase through their use in renewable-energy technologies, digital systems, electronics, aerospace, defence, specialised alloys and other strategic applications [1,2,3]. From an environmental perspective, this strengthens the rationale for generating baseline occurrence data in biological matrices that are not routinely covered by regulatory or monitoring programmes.

Although the *Critical Raw Materials Act* is not an environmental toxicology instrument, its emphasis on secure supply, diversification, circularity, recycling and environmental sustainability provides a useful policy context for expanding baseline surveillance of less-regulated technology-critical elements [4]. Such surveillance is particularly relevant because the environmental behaviour, bioavailability and biological uptake of many of these elements remain incompletely characterised. Ga and Ge show matrix-dependent behaviour and may occur at low concentrations close to analytical detection limits [3,5,6,8]. Nb and Ta are generally associated with resistant mineral phases and low environmental mobility, although measurable biological uptake has been reported under specific environmental conditions [9,10,11]. W may show greater environmental variability because tungstate mobility and uptake can be influenced by pH, organic matter, microbial activity and soil properties [13,14,16,17,18,19]. These element-specific behaviours provide a rationale for future monitoring approaches that integrate geochemical context with biological matrices.

Within this case-study context, wild mushrooms may offer a complementary biological matrix for occurrence monitoring because they integrate local substrate, taxonomic and microhabitat influences and provide biological occurrence information distinct from park-scale soil chemistry. The present data therefore contribute to an early baseline for Ga, Ge, Nb, Ta and W in UK terrestrial biota, while avoiding overinterpretation as source apportionment, food-safety assessment or direct soil-to-fruiting-body transfer. This distinction is important because the composite topsoil data used in the present study describe park-scale geochemical context, whereas the mushroom data represent fruiting-body-level biological profiles. Future monitoring should therefore avoid treating composite soil concentrations as direct predictors of individual mushroom composition unless paired microhabitat-scale substrate, rhizosphere, litter or mycelial data are available.

The strongest immediate value of the present dataset is baseline generation. As demand for technology-critical elements grows, environmental monitoring will require reference data from non-mining, urban, peri-urban and rural settings [1,3]. Wild fungi are relevant because they integrate local environmental conditions and because some taxa occur in public green spaces where human–environment interactions are common. The present data provide a starting point for Ga, Ge, Nb, Ta and W in UK wild mushrooms, highlighting both measurable occurrence and the need for careful censoring-aware reporting. This is particularly important for Ge, which was detected in only a minority of fruiting-body-level units and should therefore remain an exploratory endpoint unless lower detection limits and larger taxon-specific datasets are available.

The findings also support the inclusion of less routinely monitored and technology-related elements in exploratory multielement ICP-MS panels when sample mass and analytical resources permit. Once digestion, blank-control and instrumental workflows are established, the addition of these elements can generate useful baseline occurrence data and help identify analytical priorities for future environmental monitoring. Under the present workflow, Ga was the only element with sufficient quantitative coverage for routine continuous interpretation in wild mushrooms. Ge, Nb, Ta and W provided informative occurrence and censoring profiles, but improved analytical sensitivity, lower sample-specific detection limits and targeted sampling would be required before their routine quantitative interpretation or inclusion in continuous multielement analyses could be recommended. Overall, the results support further evaluation of wild mushrooms as exploratory, site-specific complementary biomonitoring matrices for documenting the occurrence of selected technology-critical elements. Their interpretation should remain integrated with fungal taxonomy, microhabitat information and geochemical context rather than treating fruiting bodies as stand-alone indicators of contamination, source or uptake.

### 4.6. Strengths and Limitations

The main strengths of this study are its focus on under-characterised technology-critical elements, the use of reconstructed fruiting-body-level units, explicit reporting of analytical censoring, taxon-level censoring profiles, tissue-aware interpretation, site-level composite topsoil characterisation and strengthened analytical QA/QC. The calibration and repeatability checks, together with explicit digestion-batch blank correction, strengthen the analytical traceability of the reported results, while the final censoring profiles define which elements support continuous quantitative interpretation. Ga is the principal quantitatively tractable fungal endpoint under the present analytical workflow, whereas Ge, Nb, Ta and W require censoring-aware occurrence interpretation.

The study also has limitations. Complete matrix-specific accuracy and recovery validation was not available for the full Ga–Ge–Nb–Ta–W panel. The aqueous quality-control measurements primarily documented instrumental repeatability, because their concentrations exceeded the upper calibration limits, while the available matrix reference materials did not provide acceptable target-element-specific recovery evidence for all five elements. The reported concentrations should therefore be interpreted as a blank-corrected occurrence baseline rather than as a fully matrix-validated quantitative reference dataset. Mushroom collection depended on the natural availability of eligible fruiting bodies, and taxonomic composition was unbalanced across locations, area types and urban quadrants. The rural subset was small and should not be interpreted as a county-wide rural baseline. Fruiting-body reconstruction from paired cap and stem records was not mass-weighted because complete tissue-mass data were unavailable. Ge, Nb, Ta and W showed substantial censoring, precluding robust estimation of continuous concentration distributions, multielement ordination and continuous mushroom inter-element relationships. Taxon-level patterns were additionally confounded by collection location and unequal group sizes and should therefore be regarded as descriptive. The topsoil data were based on site-level composites, which are appropriate for park-scale geochemical characterisation but not for microhabitat-scale uptake modelling. Directly paired substrate measurements, elemental speciation, fungal bioaccessibility and the labile elemental fractions potentially available to fungal mycelia were not determined. Finally, dietary exposure was not assessed because the sampling design did not represent mushrooms intended for consumption and did not provide consumption, fresh-weight, culinary-processing, bioaccessibility or speciation data required for defensible exposure estimation.

Overall, these limitations define the appropriate interpretation of the dataset as a baseline environmental-occurrence study. Wild mushrooms constitute the principal biological matrix, but their analytical usefulness differed substantially among elements. Ga supported continuous distributional interpretation, whereas Ge, Nb, Ta and W were appropriately characterised through censoring classifications and the ranges of fully quantified observations. Taxon-level patterns should be interpreted cautiously because of sampling imbalance and confounding with collection location, while the composite topsoil data provide park-scale geochemical context rather than evidence of direct uptake. Future studies should combine improved analytical sensitivity with balanced taxon-specific replication, tissue-mass recording and directly paired microhabitat-scale substrate measurements, including relevant speciation and bioavailability indicators.

## 5. Conclusions

This study provides a blank-corrected and censoring-aware environmental-occurrence baseline for Ga, Ge, Nb, Ta and W in wild mushrooms by combining fruiting-body-level reconstruction with digestion-date blank correction, analytical-portion-specific detection limits and explicit censoring classification. The baseline should be interpreted in light of incomplete target-element-specific matrix accuracy and recovery validation for the five-element panel. The reanalysis changed the evidential strength of the dataset: Ga supported continuous quantitative interpretation, whereas Ge, Nb, Ta and W were appropriately restricted to occurrence profiles and fully quantified ranges. This distinction is central to avoiding distributional, multivariate and toxicological conclusions driven by substitution or detection-limit structure.

The most robust biological result was the higher Ga concentration in stems than caps of paired *Agaricus bitorquis* fruiting bodies. Other taxon-, area- and quadrant-level patterns remain hypothesis-generating because sampling was unbalanced and fungal identity was confounded with collection location. Likewise, the 26 composite topsoil values describe park-scale acid-recoverable geochemistry but cannot support bioaccumulation factors, source attribution or direct soil-to-fungus transfer because the soil and mushroom samples were not paired at the relevant microhabitat scale.

Under the present sampling design, wild mushrooms may therefore have exploratory, site-specific value as complementary biomonitoring matrices for documenting the environmental occurrence of selected technology-critical elements, but the present findings do not support their use as universal indicators of contamination, exposure or risk. Future studies should combine improved analytical sensitivity with balanced taxon-specific replication, tissue-mass recording and directly paired fruiting-body, substrate, litter, mycelial-zone and porewater sampling. Chemical speciation, labile fractions, culinary processing, bioaccessibility, consumption patterns and toxicological dose–response information will also be required before dietary or ecological risks can be characterised quantitatively.

## Figures and Tables

**Table 1:** Blank-corrected censoring profiles and concentration summaries for Ga, Ge, Nb, Ta and W in 121 reconstructed wild-mushroom fruiting-body-level units from Leicester and Leicestershire, UK.

Element	*N*	Fully Quantified, *n* (%)	Partially Censored, *n* (%)	Fully Censored, *n* (%)	Fully Quantified Range (ng g^−1^ dw)	Median (ng g^−1^ dw)	IQR (ng g^−1^ dw)
**Ga**	121	106 (87.6)	0 (0.0)	15 (12.4)	25.51–14,410.84	475.48	278.70–941.63
**Ge**	121	16 (13.2)	0 (0.0)	105 (86.8)	3.36–45.99	—	—
**Nb**	121	33 (27.3)	7 (5.8)	81 (66.9)	3.49–256.49	—	—
**Ta**	121	10 (8.3)	4 (3.3)	107 (88.4)	4.58–50.91	—	—
**W**	121	24 (19.8)	4 (3.3)	93 (76.9)	9.79–1160.68	—	—

Fully quantified units contained no censored analytical component; partially censored units contained at least one quantified and one censored component; and fully censored units contained no quantified component. The fully quantified range includes only units classified as fully quantified and does not incorporate partially or fully censored observations. The Ga median and interquartile range (IQR) were estimated across all 121 units using regression on order statistics (ROS). Distributional estimates were not calculated for Ge, Nb, Ta or W because quantitative coverage was insufficient. Ge classifications use the conservative maximum-blank criterion selected for the primary analysis; the alternative date-specific blank-correction result (24/121 units with at least one quantified component) is reported in the text as a sensitivity analysis. dw, dry weight; —, not estimated. Percentages may not sum exactly to 100 because of rounding.

**Table 2:** Blank-corrected taxon-level censoring profiles and Ga concentration summaries for the five principal analytical groups. Concentrations are expressed as ng g^−1^ dw.

Element	FQ, *n* (%)	PC, *n* (%)	FC, *n* (%)	FQ Range	Mean ± SD	Median (IQR)
***Agaricus bitorquis* (*N* = 22)**						
**Ga**	22 (100.0)	0 (0.0)	0 (0.0)	148.26–941.63	394.15 ± 186.46	320.60 (288.57–424.28)
**Ge**	0 (0.0)	0 (0.0)	22 (100.0)	—	—	—
**Nb**	3 (13.6)	5 (22.7)	14 (63.6)	3.61–6.95	—	—
**Ta**	0 (0.0)	4 (18.2)	18 (81.8)	—	—	—
**W**	2 (9.1)	2 (9.1)	18 (81.8)	15.44–16.69	—	—
***Coprinopsis atramentaria* (*N* = 8)**						
**Ga**	7 (87.5)	0 (0.0)	1 (12.5)	241.27–6622.19	2358.89 ± 2611.16	1004.78 (347.38–4143.68)
**Ge**	2 (25.0)	0 (0.0)	6 (75.0)	7.59–30.07	—	—
**Nb**	4 (50.0)	0 (0.0)	4 (50.0)	10.68–87.41	—	—
**Ta**	0 (0.0)	0 (0.0)	8 (100.0)	—	—	—
**W**	1 (12.5)	0 (0.0)	7 (87.5)	71.27	—	—
***Mycena citrinomarginata* (*N* = 22)**						
**Ga**	21 (95.5)	0 (0.0)	1 (4.5)	549.40–7862.47	1525.47 ± 1484.45	1223.45 (886.72–1505.27)
**Ge**	1 (4.5)	0 (0.0)	21 (95.5)	45.99	—	—
**Nb**	4 (18.2)	0 (0.0)	18 (81.8)	27.98–126.57	—	—
**Ta**	1 (4.5)	0 (0.0)	21 (95.5)	50.91	—	—
**W**	2 (9.1)	0 (0.0)	20 (90.9)	31.78–98.52	—	—
***Panaeolina foenisecii* (*N* = 28)**						
**Ga**	19 (67.9)	0 (0.0)	9 (32.1)	309.25–14,410.84	1170.83 ± 2647.30	585.58 (335.35–817.26)
**Ge**	1 (3.6)	0 (0.0)	27 (96.4)	8.29	—	—
**Nb**	4 (14.3)	0 (0.0)	24 (85.7)	4.39–256.49	—	—
**Ta**	0 (0.0)	0 (0.0)	28 (100.0)	—	—	—
**W**	1 (3.6)	0 (0.0)	27 (96.4)	1160.68	—	—
**Unknown/unidentified group (*N* = 17)**						
**Ga**	16 (94.1)	0 (0.0)	1 (5.9)	72.16–1528.62	320.80 ± 345.56	220.56 (138.92–357.56)
**Ge**	6 (35.3)	0 (0.0)	11 (64.7)	3.36–29.75	—	—
**Nb**	11 (64.7)	0 (0.0)	6 (35.3)	3.49–18.30	—	—
**Ta**	9 (52.9)	0 (0.0)	8 (47.1)	4.58–25.27	—	—
**W**	14 (82.4)	0 (0.0)	3 (17.6)	9.79–35.23	—	—

**FQ**, fully quantified; **PC**, partially censored; **FC**, fully censored; **IQR**, interquartile range; **SD**, standard deviation; **dw**, dry weight. FQ ranges include only units classified as fully quantified. For Ga, the mean, SD, median and IQR for *Agaricus bitorquis* were calculated directly from the 22 fully quantified blank-corrected observations; estimates for groups containing censored Ga observations were obtained using NADA::cenros with observation-specific publication censoring bounds and the blank-corrected quantified concentrations. Distributional estimates were not calculated for Ge, Nb, Ta or W because quantitative coverage was insufficient. Single fully quantified observations are reported as individual values rather than ranges. *Coprinopsis atramentaria* is retained for descriptive transparency, but its results are tentative because the group comprised only eight units. The unknown/unidentified group may contain multiple taxa and is retained solely as an analytical category. Taxon and collection location were strongly confounded; therefore, no inferential comparisons among groups were performed. Percentages may not sum exactly to 100 because of rounding.

**Table 3:** Blank-corrected cap–stem Ga concentrations and censoring profiles for Ga, Ge, Nb, Ta and W in 22 paired *Agaricus bitorquis* fruiting bodies. Concentrations are expressed as ng g^−1^ dw.

Element	FQ Pairs, *n* (%)	PC Pairs, *n* (%)	FC Pairs, *n* (%)	Tissue	FQ Tissue Range	Mean ± SD	Median (IQR)	Wilcoxon *p*-Value	BH-FDR
**Ga**	22 (100.0)	0 (0.0)	0 (0.0)	Cap	113.70–827.90	299.58 ± 165.50	283.47 (191.80–333.68)	5.25 × 10^−5^	1.05 × 10^−4^
				Stem	139.89–1072.77	488.72 ± 235.82	457.87 (340.12–550.20)		
**Ge**	0 (0.0)	0 (0.0)	22 (100.0)	—	—	—	—	—	—
**Nb**	3 (13.6)	5 (22.7)	14 (63.6)	—	—	—	—	—	—
**Ta**	0 (0.0)	4 (18.2)	18 (81.8)	—	—	—	—	—	—
**W**	2 (9.1)	2 (9.1)	18 (81.8)	—	—	—	—	—	—

FQ, fully quantified; PC, partially censored; FC, fully censored; IQR, interquartile range; SD, standard deviation; dw, dry weight. A pair was classified as FQ when both cap and stem met the applicable detection criterion, PC when only one tissue was quantified and FC when neither tissue was quantified. Tissue-specific distributional summaries and paired inference were restricted to Ga because it was fully quantified in all 22 pairs. The Wilcoxon signed-rank test compares matched cap and stem Ga concentrations; the Benjamini–Hochberg false-discovery-rate-adjusted value is reported as BH-FDR. No tissue-specific continuous summaries or inferential tests were calculated for Ge, Nb, Ta or W because the number of fully quantified pairs was insufficient. Working LoDs were analytical-portion-specific; therefore, no single fixed LoD is shown.

**Table 4:** Site-level acid-recoverable concentrations of Ga, Ge, Nb, Ta and W in 26 composite topsoil samples from Leicester and Leicestershire. Concentrations are expressed as mg kg^−1^ dw.

Element	*N*	>LoD, *n* (%)	Censored, *n* (%)	Working LoD	Range	Mean ± SD	Geometric Mean	Median (IQR)	P95	P97.5
**Ga**	26	26 (100.0)	0 (0.0)	0.01296	28.24–369.26	72.73 ± 63.15	62.30	59.84 (50.83–70.99)	102.30	203.65
**Ge**	26	26 (100.0)	0 (0.0)	0.01130	1.102–2.014	1.413 ± 0.200	1.400	1.367 (1.292–1.488)	1.693	1.816
**Nb**	26	26 (100.0)	0 (0.0)	0.02264	0.795–1.017	0.875 ± 0.056	0.873	0.860 (0.839–0.908)	0.970	0.991
**Ta**	26	26 (100.0)	0 (0.0)	0.03377	1.0081–1.0221	1.0110 ± 0.0028	1.0110	1.0108 (1.0095–1.0117)	1.0140	1.0171
**W**	26	26 (100.0)	0 (0.0)	0.31156	0.678–0.950	0.719 ± 0.054	0.717	0.704 (0.692–0.722)	0.781	0.851

LoD, limit of detection; IQR, interquartile range; SD, standard deviation; dw, dry weight. The field programme generated one homogenised composite for each of 26 monitored locations. Each composite was processed in duplicate, and the two source records were arithmetically averaged before calculation of the site-level summaries shown here; therefore, *N* = 26 represents independent locations rather than 52 duplicate records. All site-level values were above the corresponding fixed topsoil working LoD. The range, mean, SD, geometric mean, median, IQR, P95 and P97.5 were calculated from the 26 site-level values. The concentrations represent HNO_3_/HCl acid-recoverable fractions and should not be interpreted as complete total-soil inventories, labile or biologically available concentrations, or point-level evidence of individual mushroom uptake.

## Data Availability

The data presented in this study are available in the Open Science Framework (OSF) repository, *Leicester topsoil and wild mushroom metal dataset*, at https://osf.io/xbrd7/overview?view_only=750aba9f380044e38ce5c9162e0d4020 (accessed on 14 July 2026). The repository includes curated CSV files containing raw and final calculated concentrations, limits of detection (LoDs) and below-LoD flags for the topsoil and wild mushroom datasets. The dataset is released under the Creative Commons Attribution 4.0 International licence (CC BY 4.0).

## Supplementary Materials

The following supporting information can be downloaded at https://www.mdpi.com/article/10.3390/toxics14080723/s1, Table S1, calibration, detection limits, censoring and aqueous quality-control information for Ga, Ge, Nb, Ta and W in 121 mushroom fruiting-body units and 26 composite topsoil samples; Table S2, site-level comparative geochemical screening ratios based on the pollution index (PI) and Fe-normalised enrichment factor (EF) for Ga, Ge, Nb, Ta and W in the 26 site-level composite topsoil samples; Table S3, detection and blank-corrected concentration profiles for rare or low-replication taxa not retained in main Table 2. Concentrations are reported as ng g^−1^ dw; Table S4, blank-corrected area-type censoring profiles and Ga concentration summaries for wild-mushroom fruiting-body-level units from Leicester and Leicestershire, UK; Table S5, blank-corrected censoring profiles and Ga concentration summaries across urban quadrants in Leicester, UK; Figure S1, Fe-normalised contextual EF screening-ratio distributions for Ga, Ge, Nb, Ta and W in the composite topsoil samples; Figure S2, Spearman correlation heatmap for Ga, Ge, Nb, Ta and W in the 26 site-level composite topsoil samples.
