# Supplementary material for: Occurrence and Detection Profiles of Technology-Critical Elements (Ga, Ge, Nb, Ta and W) in Wild Mushrooms from Leicester and Leicestershire, UK
Source: Toxics. 2026 Aug 14;14(8):723. doi: 10.3390/toxics14080723 (PMC13517823; doi:10.3390/toxics14080723)
Supplement: Supplementary file 1 [file toxics-14-00723-s001.zip › Supplementary Tables Ga Ge.pdf]

Table S1. Calibration, detection limits, censoring and aqueous quality-control information for Ga, Ge, Nb, Ta and W in 121 mushroom fruiting-body units and 26 composite topsoil samples.

Panel A. Calibration, detection limits and analytical coverage.

Matrix	Element	Calibration range (μg L⁻¹)	R^2 (6 d.p.)	Instrumental DL (μg L⁻¹)	Wash-based IDL, 3 × SD (μg L⁻¹)	Working LoD	Final detection profile
Mushroom	Ga	0.063–88.093	0.999959	0.07200	0.00564	31.4 (3.08–1,935) ng g⁻¹ dw	106 FQ / 0 PC / 15 FC
	Ge	0.056–77.759	0.999946	0.00600	0.00142	2.93 (0.287–181) ng g⁻¹ dw	16 FQ / 0 PC / 105 FC
	Nb	0.056–77.759	0.999944	0.00300	0.00254	3.28 (0.322–202) ng g⁻¹ dw	33 FQ / 7 PC / 81 FC
	Ta	0.056–77.759	0.998936	0.00300	0.00306	3.5 (0.343–216) ng g⁻¹ dw	10 FQ / 4 PC / 107 FC
	W	0.056–77.759	0.999670	0.01600	0.00944	9.31 (0.913–574) ng g⁻¹ dw	24 FQ / 4 PC / 93 FC
Composite topsoil	Ga	0.045–66.895	0.999331	0.01200	—	0.01296 mg kg⁻¹ dw	26/26 site-level values > LoD
	Ge	0.074–111.133	0.999511	0.00900	—	0.01130 mg kg⁻¹ dw	26/26 site-level values > LoD
	Nb	0.074–111.133	0.999552	0.00100	—	0.02264 mg kg⁻¹ dw	26/26 site-level values > LoD
	Ta	0.074–111.133	0.999163	0.00200	—	0.03377 mg kg⁻¹ dw	26/26 site-level values > LoD
	W	0.074–111.133	0.999287	0.01500	—	0.31156 mg kg⁻¹ dw	26/26 site-level values > LoD

Calibration R^2 values were calculated from the nominal concentrations and net instrumental responses of the ten calibration standards using unweighted ordinary least-squares regression with a freely fitted intercept. For the mushroom analyses, instrumental detection limits (IDLs) were calculated as three times the standard deviation (SD) of four measurements of a 2% HNO_3 wash solution. Corresponding wash measurements were unavailable for the topsoil analytical batch; therefore, wash-based IDLs could not be calculated for this matrix. Mushroom working limits of detection (LoDs) were analytical-portion-specific and are reported as the median (range) across the 153 analytical portions used to reconstruct the 121 fruiting-body-level units; topsoil working LoDs were fixed matrix-level values. FQ, fully quantified; PC, partially censored; FC, fully censored; DL, detection limit; dw, dry weight. For Ge, the primary detection profile was based on the conservative maximum-blank criterion; the corresponding date-specific sensitivity classification was 21 FQ, 3 PC and 97 FC.

Panel B. Aqueous QCA/QCB repeatability checks and calibration-range context.

Matrix	Element	QC label	Nominal original-solution concentration (μg L⁻¹)	Nominal concentration introduced after ×2 dilution (μg L⁻¹)	Upper calibration level (μg L⁻¹)	Above upper calibration level (%)	Instrument-reported concentration, mean ± SD (μg L⁻¹)	RSD (%)
Mushroom	Ga	QCA 251	251.0	125.5	88.093	42.5	215.33 ± 9.58	4.45
	Ge	QCB 280	280.0	140.0	77.759	80.0	290.01 ± 17.85	6.16
	Nb	QCB 280	280.0	140.0	77.759	80.0	291.24 ± 13.93	4.78
	Ta	QCB 280	280.0	140.0	77.759	80.0	240.74 ± 13.36	5.55
	W	QCB 280	280.0	140.0	77.759	80.0	275.05 ± 5.12	1.86
Composite topsoil	Ga	QCA 255	255.0	127.5	66.895	90.6	250.33 ± 8.59	3.43
	Ge	QCB 274	274.0	137.0	111.133	23.3	259.15 ± 3.83	1.48
	Nb	QCB 274	274.0	137.0	111.133	23.3	287.48 ± 8.63	3.00
	Ta	QCB 274	274.0	137.0	111.133	23.3	267.99 ± 3.62	1.35
	W	QCB 274	274.0	137.0	111.133	23.3	263.58 ± 15.48	5.87

QCA and QCB were aqueous quality-control (QC) solutions used for Ga and for Ge, Nb, Ta and W, respectively. Each was measured on three occasions after automated twofold dilution. The nominal concentrations introduced into the ICP–MS exceeded the corresponding upper calibration limit. The reported means and relative standard deviations (RSDs) therefore characterise repeatability under extrapolated conditions and were not used to assess within-range accuracy or analytical recovery; recovery percentages were not calculated. Instrument-reported concentrations are shown on the original-solution scale, whereas introduced concentrations account for the twofold dilution. Aqueous concentrations are expressed as $\mu g\ L^{-1}$.

Table S2. Site-level comparative geochemical screening ratios based on the pollution index (PI) and Fe-normalised enrichment factor (EF) for Ga, Ge, Nb, Ta and W in the 26 site-level composite topsoil samples.

Panel A. PI-based comparative geochemical screening ratios.

Site code	Site name	Area	Quadrant	PI (Ga)	PI (Ge)	PI (Nb)	PI (Ta)	PI (W)
AMNR	Aylestone Meadows Nature Reserve	Urban	SW	9.635	1.559	0.081	1.460	0.452
AP	Abbey Park	Urban	NE	3.205	1.185	0.075	1.440	0.353
APF	Aylestone Playing Fields	Urban	SW	10.429	2.165	0.071	1.444	0.344
ARG	Aylestone Recreation Ground	Urban	SE	5.865	1.551	0.073	1.442	0.333
ASNR	Alterstones Nature Reserve	Rural	NW	2.852	1.614	0.085	1.444	0.377
AppP	Appleton Park	Urban	NE	4.903	1.323	0.071	1.442	0.331
BHCP	Brocks Hill Country Park	Rural	SE	5.147	1.501	0.068	1.448	0.326
BP	Bede Park	Urban	SW	5.224	1.441	0.070	1.442	0.326
BdP	Bradgate Park	Rural	NW	5.062	1.443	0.077	1.444	0.352
BeauP	Beaumont Park	Urban	NW	36.926	1.341	0.071	1.440	0.336
BrP	Braunstone Park	Urban	SW	6.082	1.485	0.070	1.442	0.327
CG	Castle Gardens	Urban	NW	7.593	1.824	0.075	1.444	0.339
CMNR	Cossington Meadows Nature Reserve	Rural	NE	2.824	1.199	0.072	1.445	0.334
CP	Croft Pasture	Rural	SW	5.957	1.388	0.071	1.444	0.331
HP	Humberstone Park	Urban	NE	6.847	1.808	0.069	1.445	0.331
KnP	Knighton Park	Urban	SE	8.598	1.395	0.072	1.445	0.330
MM	Mossdale Meadows	Urban	SW	5.876	1.366	0.066	1.442	0.323
NB	Narborough Bog	Rural	SW	7.183	1.790	0.079	1.447	0.343
NMP	Nelson Mandela Park	Urban	SE	6.010	1.648	0.078	1.447	0.351
SHP	Spinney Hill Park	Urban	NE	6.769	1.749	0.067	1.442	0.329
SL	Syston Lakes	Rural	NE	7.847	1.544	0.069	1.449	0.358
SWP	Stokes Wood Park	Urban	NW	6.575	1.454	0.076	1.440	0.344
TMP	The Miles Piece	Rural	NE	5.737	1.395	0.067	1.446	0.323
VP	Victoria Park	Urban	SE	4.842	1.551	0.074	1.444	0.339
WMP	Watermead Park	Urban	NE	6.298	1.397	0.070	1.442	0.328
WP	Western Park	Urban	NW	4.823	1.385	0.077	1.440	0.339

Panel B. Fe-normalised EF-based comparative geochemical screening ratios.

Site code	Site name	Area	Quadrant	Fe (mg kg^{-1} dw)	EF (Ga)	EF (Ge)	EF (Nb)	EF (Ta)	EF (W)
AMNR	Aylestone Meadows Nature Reserve	Urban	SW	38432.5	7.020	1.135	0.059	1.064	0.330
AP	Abbey Park	Urban	NE	22485.0	3.992	1.475	0.093	1.794	0.439
APF	Aylestone Playing Fields	Urban	SW	34465.0	8.472	1.759	0.058	1.173	0.279
ARG	Aylestone Recreation Ground	Urban	SE	34192.5	4.802	1.270	0.059	1.181	0.273
ASNR	Alterstones Nature Reserve	Rural	NW	19522.5	4.091	2.315	0.121	2.071	0.540
AppP	Appleton Park	Urban	NE	34895.0	3.934	1.062	0.057	1.157	0.265
BHCP	Brocks Hill Country Park	Rural	SE	34495.0	4.178	1.218	0.056	1.175	0.264
BP	Bede Park	Urban	SW	26575.0	5.504	1.518	0.074	1.520	0.344
BdP	Bradgate Park	Rural	NW	24050.0	5.893	1.680	0.090	1.682	0.410
BeauP	Beaumont Park	Urban	NW	19270.0	53.655	1.948	0.103	2.093	0.488
BrP	Braunstone Park	Urban	SW	23770.0	7.164	1.749	0.083	1.699	0.385
CG	Castle Gardens	Urban	NW	27350.0	7.773	1.868	0.077	1.478	0.347
CMNR	Cossington Meadows Nature Reserve	Rural	NE	32682.5	2.419	1.028	0.062	1.238	0.286
CP	Croft Pasture	Rural	SW	33682.5	4.952	1.154	0.059	1.201	0.276
HP	Humberstone Park	Urban	NE	39540.0	4.849	1.281	0.049	1.024	0.235
KnP	Knighton Park	Urban	SE	32802.5	7.340	1.190	0.062	1.233	0.281
MM	Mossdale Meadows	Urban	SW	26822.5	6.134	1.426	0.069	1.505	0.337
NB	Narborough Bog	Rural	SW	31635.0	6.358	1.584	0.070	1.280	0.303
NMP	Nelson Mandela Park	Urban	SE	28955.0	5.812	1.594	0.075	1.399	0.339
SHP	Spinney Hill Park	Urban	NE	26507.5	7.150	1.848	0.070	1.523	0.348
SL	Syston Lakes	Rural	NE	41900.0	5.244	1.032	0.046	0.968	0.239
SWP	Stokes Wood Park	Urban	NW	22257.5	8.271	1.829	0.095	1.812	0.433
TMP	The Miles Piece	Rural	NE	34485.0	4.658	1.132	0.054	1.174	0.263
VP	Victoria Park	Urban	SE	24485.0	5.537	1.774	0.084	1.651	0.388
WMP	Watermead Park	Urban	NE	24587.5	7.172	1.591	0.080	1.642	0.374
WP	Western Park	Urban	NW	19660.0	6.869	1.973	0.110	2.051	0.482

PI was calculated as the element concentration in each sample divided by the corresponding Atlas median. EF was calculated as the element/Fe ratio in each sample divided by the element/Fe ratio in the Atlas reference. Atlas reference medians were 10 mg kg^{-1} for Ga, 0.93 mg kg^{-1} for Ge, 12 mg kg^{-1} for Nb, 0.70 mg kg^{-1} for Ta, 2.1 mg kg^{-1} for W and 28,000 mg kg^{-1} for Fe. Study concentrations represent HNO_3/HCl acid-recoverable fractions, whereas the Atlas medians were generated using a different national-scale sampling and analytical framework; the study and reference values are therefore not method-equivalent. PI and EF values are provided exclusively as comparative geochemical screening ratios and should not be interpreted as formal pollution or enrichment classifications, regulatory exceedances, estimates of bioavailability, source attribution or evidence of soil-to-fungus transfer. Fe concentrations were measured in the same 26 composite topsoil samples [33]; Atlas reference values were obtained from [48].

Table S3. Detection and blank-corrected concentration profiles for rare or low-replication taxa not retained in main Table 2. Concentrations are reported as ng g⁻¹ dw.

Taxon	Element	*N*	Fully quantified, *n*	Partially censored, *n*	Fully censored, *n*	At least one component quantified, *n* (%)	Fully quantified value or range (ng g⁻¹ dw)
Agaricus arvensis	Ga	2	2	0	0	2 (100.0)	140–269
	Ge	2	0	0	2	0 (0.0)	—
	Nb	2	0	1	1	1 (50.0)	—
	Ta	2	0	0	2	0 (0.0)	—
	W	2	0	1	1	1 (50.0)	—
Agaricus nivescens	Ga	1	1	0	0	1 (100.0)	100
	Ge	1	0	0	1	0 (0.0)	—
	Nb	1	0	0	1	0 (0.0)	—
	Ta	1	0	0	1	0 (0.0)	—
	W	1	0	0	1	0 (0.0)	—
Armillaria mellea	Ga	3	2	0	1	2 (66.7)	25.5–133
	Ge	3	0	0	3	0 (0.0)	—
	Nb	3	0	0	3	0 (0.0)	—
	Ta	3	0	0	3	0 (0.0)	—
	W	3	0	0	3	0 (0.0)	—
Hypholoma fasciculare	Ga	1	1	0	0	1 (100.0)	894
	Ge	1	1	0	0	1 (100.0)	10.4
	Nb	1	1	0	0	1 (100.0)	7.56
	Ta	1	0	0	1	0 (0.0)	—
	W	1	1	0	0	1 (100.0)	10.4
Leucoagaricus leucothites	Ga	1	1	0	0	1 (100.0)	461
	Ge	1	0	0	1	0 (0.0)	—
	Nb	1	1	0	0	1 (100.0)	3.6
	Ta	1	0	0	1	0 (0.0)	—
	W	1	0	0	1	0 (0.0)	—
Marasmius oreades	Ga	4	4	0	0	4 (100.0)	234–1,480
	Ge	4	3	0	1	3 (75.0)	4.62–17.8
	Nb	4	2	0	2	2 (50.0)	9.63–12.2
	Ta	4	0	0	4	0 (0.0)	—
	W	4	2	0	2	2 (50.0)	11.8–15.6
Pholiota adiposa	Ga	2	2	0	0	2 (100.0)	373–502
	Ge	2	0	0	2	0 (0.0)	—

Taxon	Element	*N*	Fully quantified, *n*	Partially censored, *n*	Fully censored, *n*	At least one component quantified, *n* (%)	Fully quantified value or range (ng g^{-1} dw)
	Nb	2	0	0	2	0 (0.0)	—
	Ta	2	0	0	2	0 (0.0)	—
	W	2	0	1	1	1 (50.0)	—
Pholiota gummosa	Ga	1	1	0	0	1 (100.0)	1,490
	Ge	1	0	0	1	0 (0.0)	—
	Nb	1	0	0	1	0 (0.0)	—
	Ta	1	0	0	1	0 (0.0)	—
	W	1	0	0	1	0 (0.0)	—
Psathyrella corrugis	Ga	1	1	0	0	1 (100.0)	3,530
	Ge	1	1	0	0	1 (100.0)	19.4
	Nb	1	1	0	0	1 (100.0)	22.4
	Ta	1	0	0	1	0 (0.0)	—
	W	1	0	0	1	0 (0.0)	—
Psathyrella fatua	Ga	3	3	0	0	3 (100.0)	521–1,700
	Ge	3	1	0	2	1 (33.3)	11.4
	Nb	3	2	0	1	2 (66.7)	11.7–16.4
	Ta	3	0	0	3	0 (0.0)	—
	W	3	1	0	2	1 (33.3)	29.9
Psathyrella sublatispora	Ga	1	0	0	1	0 (0.0)	—
	Ge	1	0	0	1	0 (0.0)	—
	Nb	1	0	0	1	0 (0.0)	—
	Ta	1	0	0	1	0 (0.0)	—
	W	1	0	0	1	0 (0.0)	—
Vascellum pratense	Ga	2	1	0	1	1 (50.0)	291
	Ge	2	0	0	2	0 (0.0)	—
	Nb	2	0	0	2	0 (0.0)	—
	Ta	2	0	0	2	0 (0.0)	—
	W	2	0	0	2	0 (0.0)	—
Xerocomus porosporus	Ga	2	2	0	0	2 (100.0)	527–774
	Ge	2	0	0	2	0 (0.0)	—
	Nb	2	0	1	1	1 (50.0)	—
	Ta	2	0	0	2	0 (0.0)	—
	W	2	0	0	2	0 (0.0)	—

Rare and low-replication taxa are retained to document the composition of the analytical dataset and preserve valid chemical observations. These entries are descriptive only and should not be used for taxon-level inference. For taxa represented by a single fruiting-body-level unit (N = 1), only the detection or censoring classification and the single fully quantified concentration, where available, are reported; measures of dispersion and percentiles were not calculated. For elements affected by censoring, results are restricted to censoring classes and fully quantified values or ranges, without substitution for values below the applicable detection limit. — = indicates that no fruiting-body-level unit was fully quantified. The Unknown/unidentified analytical group is reported in main Table 2 and is therefore omitted from this supplementary table.

Table S4. Blank-corrected area-type censoring profiles and Ga concentration summaries for wild-mushroom fruiting-body-level units from Leicester and Leicestershire, UK.

Panel A. Element-specific analytical coverage and fully quantified concentration ranges.

Area type	Element	*N*	FQ, *n* (%)	PC, *n* (%)	FC, *n* (%)	At least one quantified, *n* (%)	Fully quantified value or range (ng g^{-1} dw)
Urban	Ga	113	99 (87.6)	0 (0.0)	14 (12.4)	99 (87.6)	25.51–14,410.84
	Ge	113	14 (12.4)	0 (0.0)	99 (87.6)	14 (12.4)	3.36–45.99
	Nb	113	29 (25.7)	7 (6.2)	77 (68.1)	36 (31.9)	3.49–256.49
	Ta	113	10 (8.8)	4 (3.5)	99 (87.6)	14 (12.4)	4.58–50.91
	W	113	23 (20.4)	4 (3.5)	86 (76.1)	27 (23.9)	9.79–1,160.68
Rural	Ga	8	7 (87.5)	0 (0.0)	1 (12.5)	7 (87.5)	241.27–6,622.19
	Ge	8	2 (25.0)	0 (0.0)	6 (75.0)	2 (25.0)	7.59–30.07
	Nb	8	4 (50.0)	0 (0.0)	4 (50.0)	4 (50.0)	10.68–87.41
	Ta	8	0 (0.0)	0 (0.0)	8 (100.0)	0 (0.0)	—
	W	8	1 (12.5)	0 (0.0)	7 (87.5)	1 (12.5)	71.27

FQ, fully quantified; PC, partially censored; FC, fully censored; dw, dry weight. “At least one quantified” comprises FQ + PC units. Fully quantified values or ranges include only units classified as FQ and do not incorporate partially or fully censored observations. Ge classifications use the conservative maximum-blank criterion selected for the primary analysis. Percentages may not sum exactly to 100 because of rounding.

Panel B. Ga distributional summaries.

Area type	N	FQ, n (%)	FC, n (%)	Fully quantified range (ng g^{-1} dw)	Mean ± SD (ng g^{-1} dw)	Median (IQR) (ng g^{-1} dw)
Urban	113	99 (87.6)	14 (12.4)	25.51–14,410.84	853.61 ± 1,566.97	464.36 (276.32–910.69)
Rural	8	7 (87.5)	1 (12.5)	241.27–6,622.19	2,358.89 ± 2,611.16	1,004.78 (347.38–4,143.68)

Ga means, standard deviations, medians and interquartile ranges were estimated across all units in each area type using regression on order statistics (ROS) with observation-specific censoring bounds and blank-corrected quantified concentrations. The fully quantified range includes only FQ observations. Continuous summaries were not calculated for Ge, Nb, Ta or W because quantitative coverage was insufficient.

Panel C. Sampling composition relevant to interpretation.

Area type	N	Collection locations, n	Analytical groups, n	Taxonomic composition
Urban	113	12	17	*Panaeolina foenisecii* (28); *Mycena citrinomarginata* (22); *Agaricus bitorquis* (22); unknown/unidentified (17); 13 additional taxa (24 units)
Rural	8	1	1	*Coprinopsis atramentaria* (8; all collected at Bradgate Park)

The rural subset comprised only eight *Coprinopsis atramentaria* units collected at one location. Area type, taxonomic composition and collection location were therefore strongly confounded, and the table is intended for descriptive occurrence reporting rather than controlled urban–rural inference.

Table S5. Blank-corrected censoring profiles and Ga concentration summaries across urban quadrants in Leicester, UK.

Panel A. Element-specific analytical coverage and fully quantified concentration ranges.

Urban quadrant	Element	*N*	FQ, *n* (%)	PC, *n* (%)	FC, *n* (%)	At least one quantified, *n* (%)	Fully quantified value or range (ng g^{-1} dw)
NE	Ga	30	21 (70.0)	0 (0.0)	9 (30.0)	21 (70.0)	100.24–14,410.84
	Ge	30	4 (13.3)	0 (0.0)	26 (86.7)	4 (13.3)	4.62–17.77
	Nb	30	6 (20.0)	0 (0.0)	24 (80.0)	6 (20.0)	4.39–256.49
	Ta	30	0 (0.0)	0 (0.0)	30 (100.0)	0 (0.0)	—
	W	30	2 (6.7)	0 (0.0)	28 (93.3)	2 (6.7)	11.80–15.63
NW	Ga	51	49 (96.1)	0 (0.0)	2 (3.9)	49 (96.1)	72.16–3,530.86
	Ge	51	8 (15.7)	0 (0.0)	43 (84.3)	8 (15.7)	3.36–29.75
	Nb	51	17 (33.3)	7 (13.7)	27 (52.9)	24 (47.1)	3.49–22.38
	Ta	51	9 (17.6)	4 (7.8)	38 (74.5)	13 (25.5)	4.58–25.27
	W	51	17 (33.3)	4 (7.8)	30 (58.8)	21 (41.2)	9.79–35.23
SE	Ga	27	25 (92.6)	0 (0.0)	2 (7.4)	25 (92.6)	25.51–7,862.47
	Ge	27	2 (7.4)	0 (0.0)	25 (92.6)	2 (7.4)	10.45–45.99
	Nb	27	6 (22.2)	0 (0.0)	21 (77.8)	6 (22.2)	3.60–126.57
	Ta	27	1 (3.7)	0 (0.0)	26 (96.3)	1 (3.7)	50.91
	W	27	3 (11.1)	0 (0.0)	24 (88.9)	3 (11.1)	10.43–98.52
SW	Ga	5	4 (80.0)	0 (0.0)	1 (20.0)	4 (80.0)	309.25–2,406.97
	Ge	5	0 (0.0)	0 (0.0)	5 (100.0)	0 (0.0)	—
	Nb	5	0 (0.0)	0 (0.0)	5 (100.0)	0 (0.0)	—
	Ta	5	0 (0.0)	0 (0.0)	5 (100.0)	0 (0.0)	—
	W	5	1 (20.0)	0 (0.0)	4 (80.0)	1 (20.0)	1,160.68

Only urban fruiting-body-level units are included. FQ, fully quantified; PC, partially censored; FC, fully censored; dw, dry weight. "At least one quantified" comprises FQ + PC units. Fully quantified values or ranges include only FQ observations. Ge classifications use the conservative maximum-blank criterion selected for the primary analysis. Percentages may not sum exactly to 100 because of rounding.

Panel B. Ga distributional summaries.

Urban quadrant	*N*	FQ, *n* (%)	FC, *n* (%)	Fully quantified range (ng g^{-1} dw)	Mean ± SD (ng g^{-1} dw)	Median (IQR) (ng g^{-1} dw)
NE	30	21 (70.0)	9 (30.0)	100.24–14,410.84	1,079.73 ± 2,560.80	565.66 (253.32–837.99)
NW	51	49 (96.1)	2 (3.9)	72.16–3,530.86	495.32 ± 558.00	322.38 (249.90–511.67)
SE	27	25 (92.6)	2 (7.4)	25.51–7,862.47	1,290.39 ± 1,425.43	922.92 (768.85–1,486.33)
SW	5	4 (80.0)	1 (20.0)	309.25–2,406.97	878.65 ± 861.83	572.96 (505.86–598.21)

Ga means, standard deviations, medians and interquartile ranges were estimated across all units within each urban quadrant using ROS with observation-specific censoring bounds and blank-corrected quantified concentrations. Continuous summaries were not calculated for Ge, Nb, Ta or W because quantitative coverage was insufficient. The SW estimates are particularly unstable because only five units were available.

Panel C. Sampling composition relevant to interpretation.

Urban quadrant	N	Collection locations, n	Analytical groups, n	Taxonomic composition
NE	30	2	4	*Panaeolina foenisecii* (23); *Marasmius oreades* (4); *Vascellum pratense* (2); *Agaricus nivescens* (1)
NW	51	6	9	*Agaricus bitorquis* (22); unknown/unidentified (17); seven additional taxa (12 units)
SE	27	3	4	*Mycena citrinomarginata* (22); *Armillaria mellea* (3); *Hypholoma fasciculare* (1); *Leucoagaricus leucothites* (1)
SW	5	1	1	*Panaeolina foenisecii* (5; all collected at Braunstone Park)

Taxonomic composition differed markedly among quadrants: NE was dominated by *Panaeolina foenisecii*, NW by *Agaricus bitorquis* and the unknown/unidentified analytical group, SE by *Mycena citrinomarginata*, and SW consisted exclusively of *Panaeolina foenisecii* from one park. Quadrant patterns therefore cannot be separated from taxonomy, collection location or fruiting-body availability and should not be interpreted as controlled spatial gradients.
