# Supplementary material for: Occurrence and Detection Profiles of Technology-Critical Elements (Ga, Ge, Nb, Ta and W) in Wild Mushrooms from Leicester and Leicestershire, UK
Source: Toxics. 2026 Aug 14;14(8):723. doi: 10.3390/toxics14080723 (PMC13517823; doi:10.3390/toxics14080723)

Figure S1. Fe-normalised contextual enrichment-factor (EF) screening-ratio distributions for Ga, Ge, Nb, Ta and W in the 26 independent site-level composite topsoil values from Leicester and Leicestershire. Each point represents one site-level value obtained after averaging the two source records from the corresponding homogenised composite. EF was calculated as $(C_{element}/C_{Fe})$sample/$(C_{element}/C_{Fe})$Atlas median using national median reference values from the *Advanced Soil Geochemical Atlas of England and Wales* [48]. The dashed line indicates EF = 1. Study concentrations represent HNO_3/HCl acid-recoverable fractions and are not method-equivalent to the Atlas medians. The figure is provided exclusively for comparative park-scale geochemical screening and should not be interpreted as a formal pollution or enrichment classification, regulatory exceedance, risk assessment, source-attribution result, estimate of bioavailability or evidence of soil-to-fungus transfer.

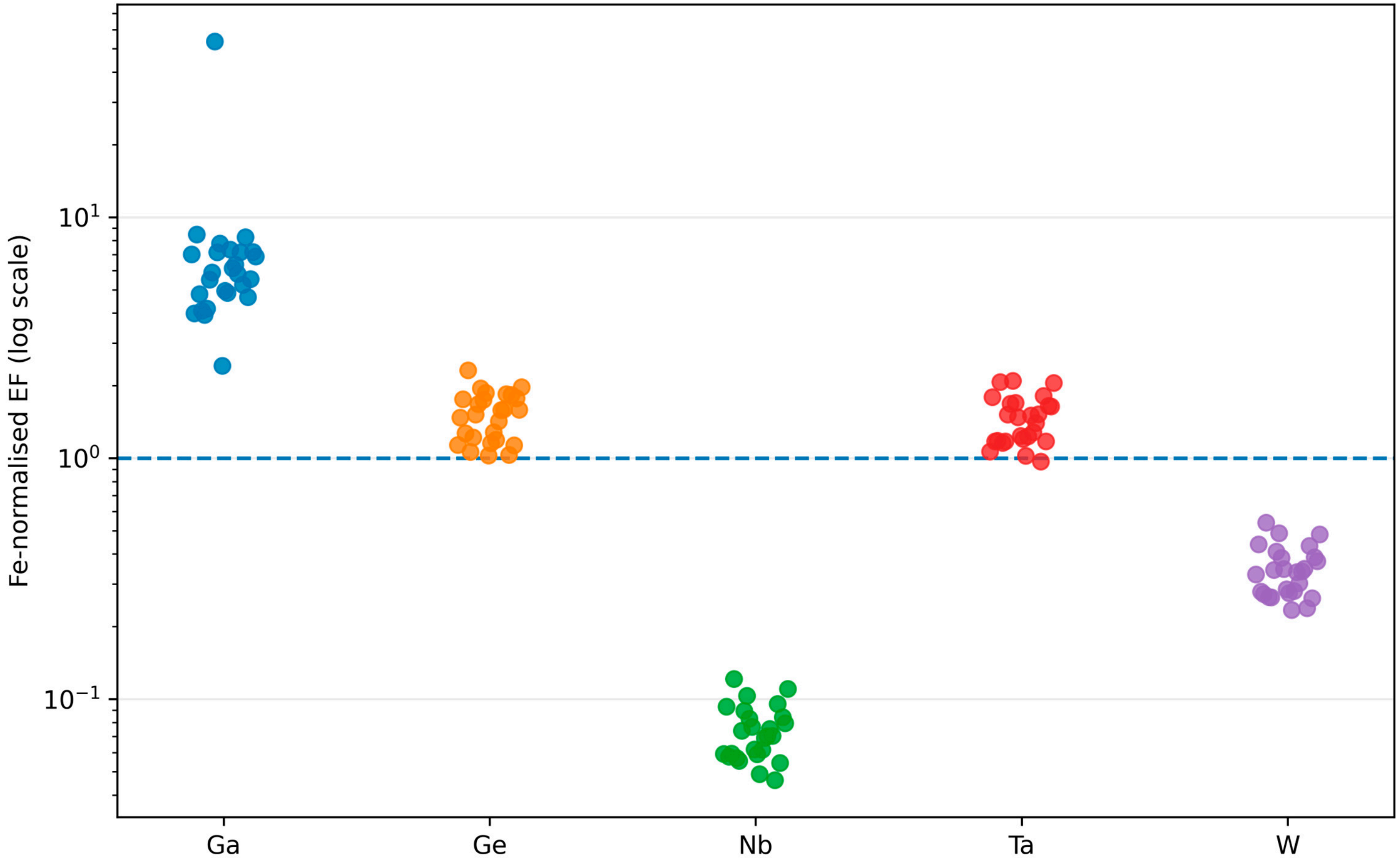

Contextual only: study HNO3/HCl acid-recoverable concentrations and Atlas national medians are not method-equivalent.

Figure S2. Spearman correlation matrix for Ga, Ge, Nb, Ta and W in the 26 independent site-level composite topsoil values from Leicester and Leicestershire. Cells display Spearman's ρ coefficients. An asterisk indicates a correlation meeting the BH-FDR-adjusted significance criterion of $q < 0.05$. The two source records from each homogenised composite were averaged before analysis. The figure describes park-scale covariation among acid-recoverable topsoil concentrations and was not used to infer source attribution, mushroom uptake, selective fungal accumulation or soil-to-fungus transfer.

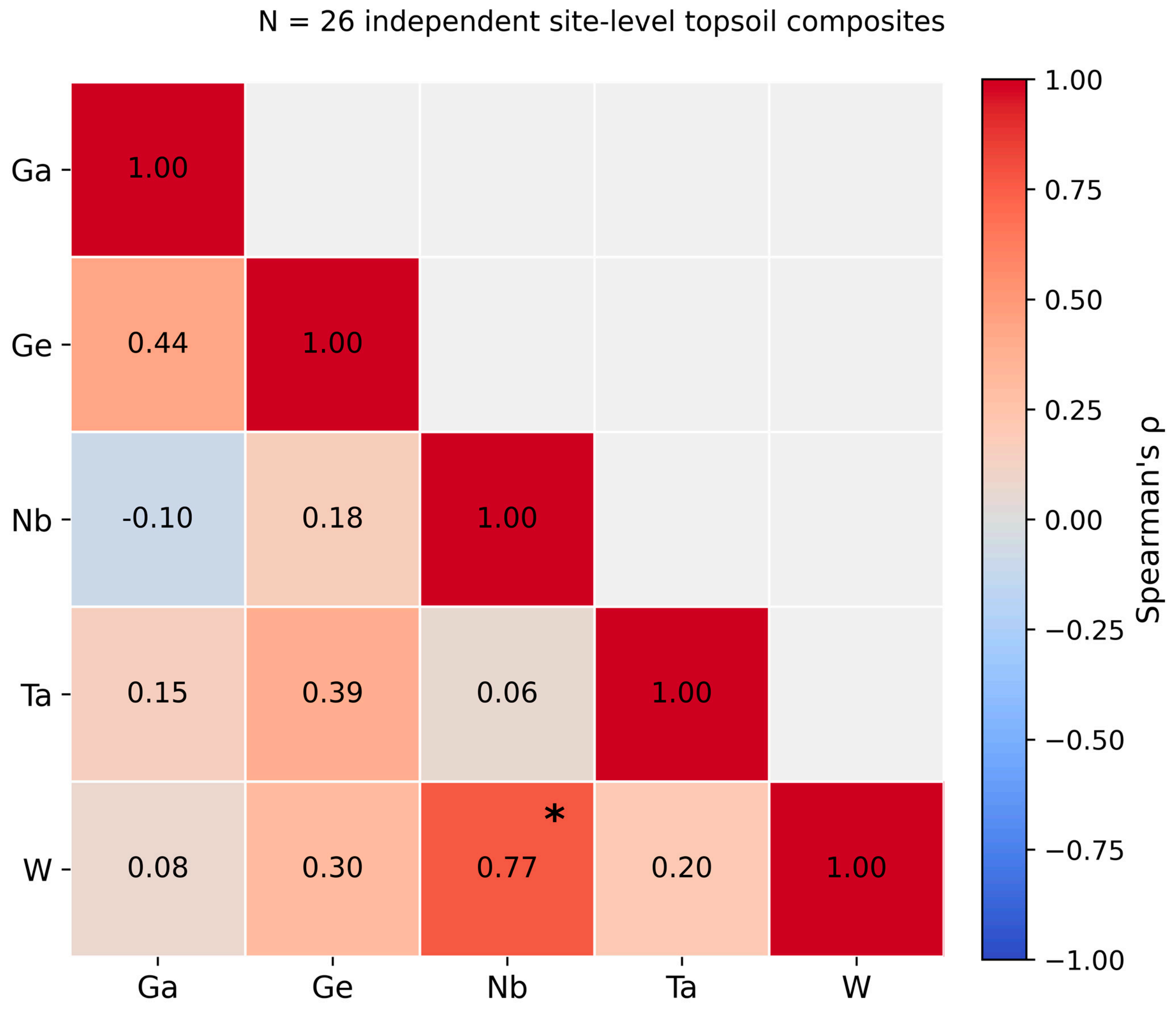

Supplement: Supplementary file 1 [file toxics-14-00723-s001.zip › Supplementary Figures Ga Ge.pdf]
